# Induction of Paraptosis by Cyclometalated Iridium Complex-Peptide Hybrids and CGP37157 via a Mitochondrial Ca^2+^ Overload Triggered by Membrane Fusion between Mitochondria and the Endoplasmic Reticulum

**DOI:** 10.1021/acs.biochem.2c00061

**Published:** 2022-04-01

**Authors:** Kenta Yokoi, Kohei Yamaguchi, Masakazu Umezawa, Koji Tsuchiya, Shin Aoki

**Affiliations:** † Faculty of Pharmaceutical Sciences, Tokyo University of Science, 2641 Yamazaki, Noda, Chiba 278-8510, Japan; ‡ Research Institute for Science and Technology, Tokyo University of Science, 2641 Yamazaki, Noda, Chiba 278-8510, Japan; § Research Institute for Biomedical Science (RIBS), Tokyo University of Science, 2641 Yamazaki, Noda, Chiba 278-8510, Japan

## Abstract

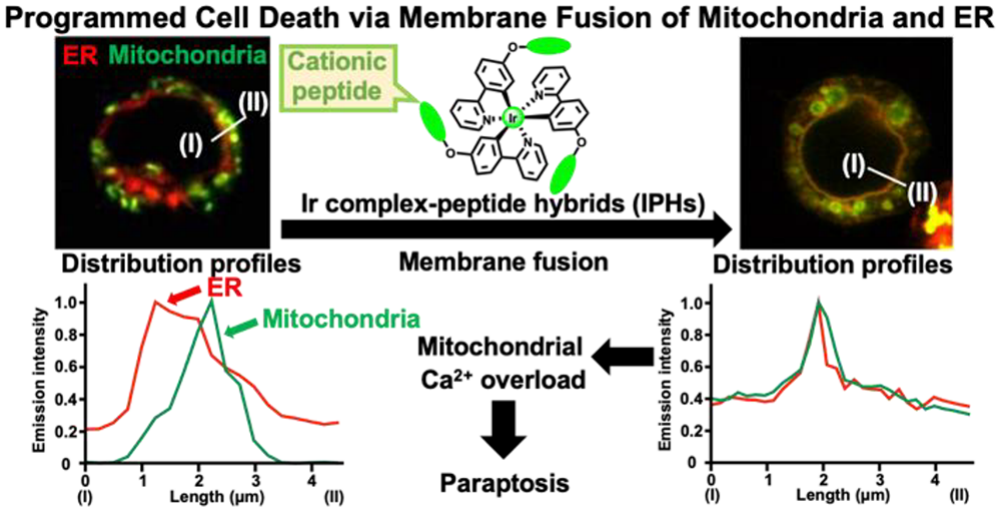

We previously reported that a cyclometalated iridium (Ir) complex-peptide hybrid (IPH) **4** functionalized with a cationic KKKGG peptide unit on the 2-phenylpyridine ligand induces paraptosis, a relatively newly found programmed cell death, in cancer cells (Jurkat cells) via the direct transport of calcium (Ca^2+^) from the endoplasmic reticulum (ER) to mitochondria. Here, we describe that CGP37157, an inhibitor of a mitochondrial sodium (Na^+^)/Ca^2+^ exchanger, induces paraptosis in Jurkat cells via intracellular pathways similar to those induced by **4**. The findings allow us to suggest that the induction of paraptosis by **4** and CGP37157 is associated with membrane fusion between mitochondria and the ER, subsequent Ca^2+^ influx from the ER to mitochondria, and a decrease in the mitochondrial membrane potential (*ΔΨ*_m_). On the contrary, celastrol, a naturally occurring triterpenoid that had been reported as a paraptosis inducer in cancer cells, negligibly induces mitochondria-ER membrane fusion. Consequently, we conclude that the paraptosis induced by **4** and CGP37157 (termed paraptosis II herein) proceeds via a signaling pathway different from that of the previously known paraptosis induced by celastrol, a process that negligibly involves membrane fusion between mitochondria and the ER (termed paraptosis I herein).

Programmed cell death (PCD) is an essential mechanism for the control of intracellular homeostasis for cell survival and proliferation and is also recognized as a cellular suicide, which is one of the strategies for anticancer therapeutics.^1^ Apoptosis, necroptosis, and autophagy are the three well-known categories of PCD and are classified by their morphological and physiological features.^2−7^ Alternative PCD types such as paraptosis,^8,9^ pyroptosis,^10^ and ferroptosis^11^ have recently been reported and have attracted considerable interest as a potential new target to eliminate drug-resistant cancer. Among them, paraptosis is a relatively new type of non-apoptotic PCD, in which cytoplasm and intracellular organelles undergo vacuolization by the dilation of mitochondria and/or the endoplasmic reticulum (ER),^12^ possibly due to the transfer of calcium (Ca^2+^) from the ER to mitochondria.^13^ Although various inducers of paraptosis, including viruses,^14^ natural products,^15−23^ organic molecules,^24,25^ and metal complexes,^26−34^ have been reported, the mechanisms responsible for this process are complicated and remain unclear. It is assumed that there are some different intracellular pathways in paraptosis, which are stimulated by these different types of paraptosis inducers, and that paraptosis could be classified into some different types.

Cyclometalated iridium (Ir) complexes such as *fac*-Ir(tpy)_3_**1a** [tpy = 2-(4′-tolyl)pyridine] and *fac*-Ir(ppy)_3_**1b** (ppy = 2-phenylpyridine) have high photophysical properties that include long Stokes shifts, high quantum yields, and long emission lifetimes.^35−37^ Therefore, Ir complexes have been proposed as attractive candidates as phosphorescent materials such as in organic light-emitting diodes (OLEDs),^38,39^ photoredox catalysts,^40,41^ bioimaging probes,^42−47^ anticancer agents,^48,49^ and related tools. We previously reported Ir complex-peptide hybrids (IPHs) that possess H_2_N-KK(K)GG peptide sequences (K, lysine; G, glycine) at the 5′- or 4′-position (the *para* or *meta* position with respect to the C–Ir bond) of the ligands, **2** and **3** or **4**, respectively (Chart 1).^50−59^ These IPHs induce cell death in Jurkat cells (T-lymphocyte leukemia) with EC_50_ (half-maximal effective concentration) values of 1.5–16 μM and are less toxic against IMR90 cells (human Caucasian fetal lung fibroblasts), which were used as a normal cell line. In addition, the luminescence emission of IPHs is enhanced in dead cells, suggesting that IPHs function as not only PCD inducers in cancer cells but also detectors of dead cells.^50−59^ The mechanistic study strongly suggested that **2c**, **3b**, **3c**, and **4** induce paraptotic cell death in Jurkat cells via a mitochondrial Ca^2+^ overload, the decrease in mitochondrial membrane potential (*ΔΨ*_m_), and cytoplasmic and mitochondrial vacuolization.^56−58^ The findings also suggested the direct transfer of Ca^2+^ into mitochondria from the ER, an intracellular Ca^2+^-storing organelle, during the paraptosis processes.

**Chart 1:**
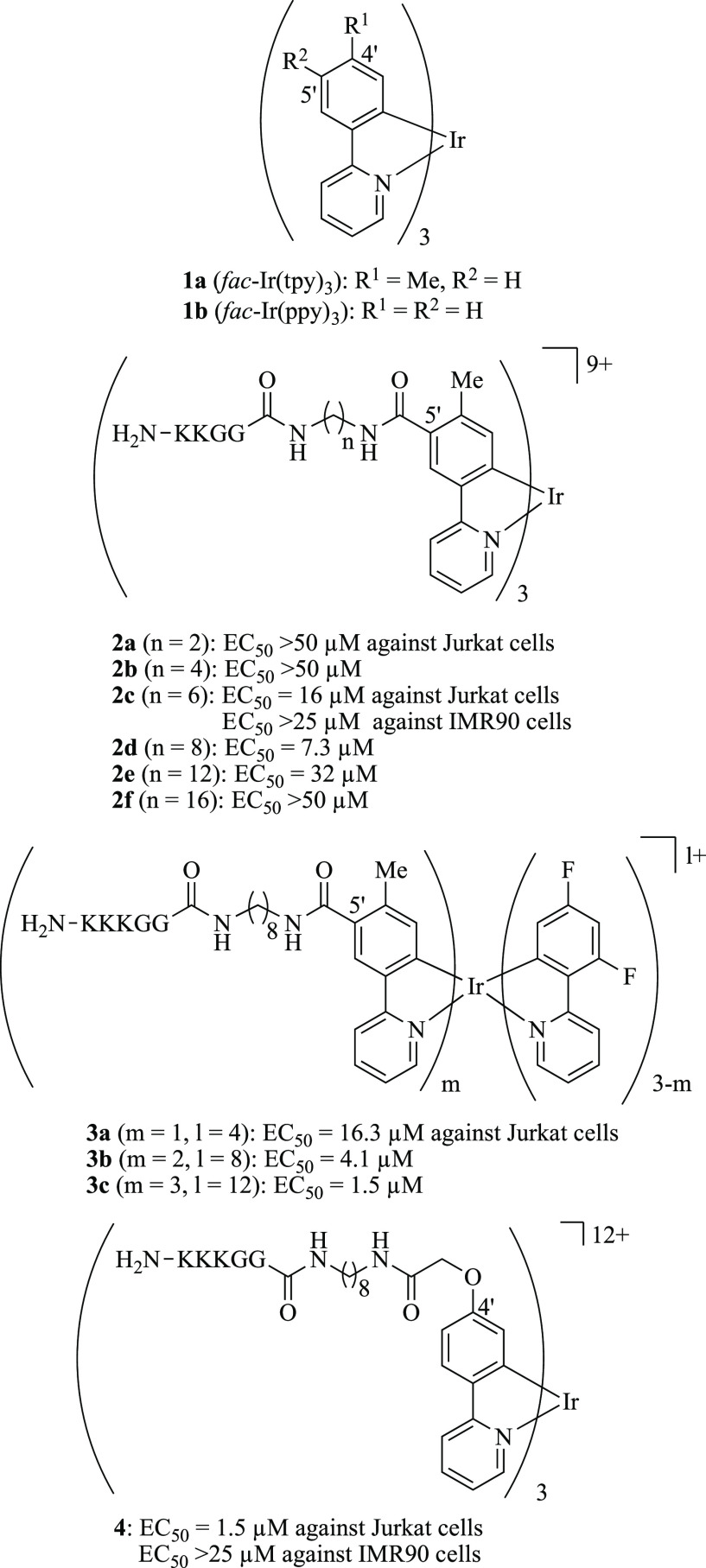
Chemical Structures of Iridium(III) Complex-Peptide Hybrids That Induce Paraptotic Cell Death

Herein, we report on the results of more detailed mechanistic studies of paraptosis induced by IPH **4**, which had the lowest EC_50_ value among **2–4** against Jurkat cells, focusing on the direct influx of Ca^2+^ from the ER into mitochondria. The findings indicate that IPH **4** is transferred to mitochondria,^56,57^ where it induces membrane fusion between mitochondria and the ER and mediates the direct influx of Ca^2+^ into mitochondria from the ER, resulting in the induction of paraptosis. It had been reported that CGP37157 is an inhibitor of the sodium (Na^+^)/Ca^2+^ exchanger (mNCX) on the outer membrane of mitochondria^60,61^ and that it affects the mitochondrial Ca^2+^ concentration (Chart 2).^62,63^ Therefore, we tested the cytotoxicity of CGP37157 against Jurkat cells and found that this compound also induces paraptosis in Jurkat cells via similar intracellular pathways to those induced by **4**. The mechanism of paraptosis induced by **4** and CGP37157 was compared with that induced by celastrol, which is a naturally occurring triterpenoid isolated from *Tripterygium wilfordii* and has also been reported to be a paraptosis inducer (Chart 2).^17,18,56−58^ We conclude that the mechanism for the paraptosis induced by both **4** and CGP37157 includes membrane fusion between mitochondria and the ER, while in the case of celastrol, such a function is negligible.

**Chart 2:**
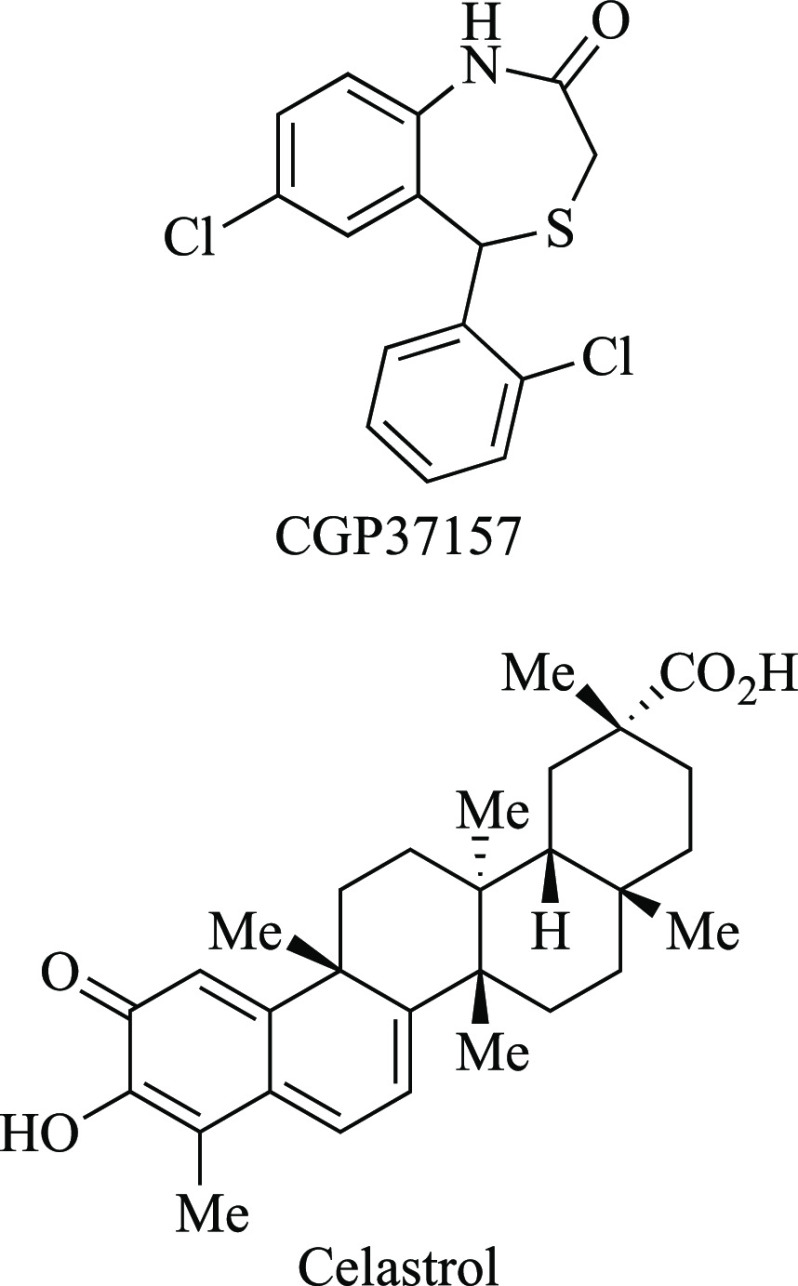
Chemical Structures of CGP37157 and Celastrol

## Results and Discussion

### Cytotoxicity of 4 against Jurkat Cells, as Evaluated by MTT Assays and Microscopic Observations

**Figure 1:**
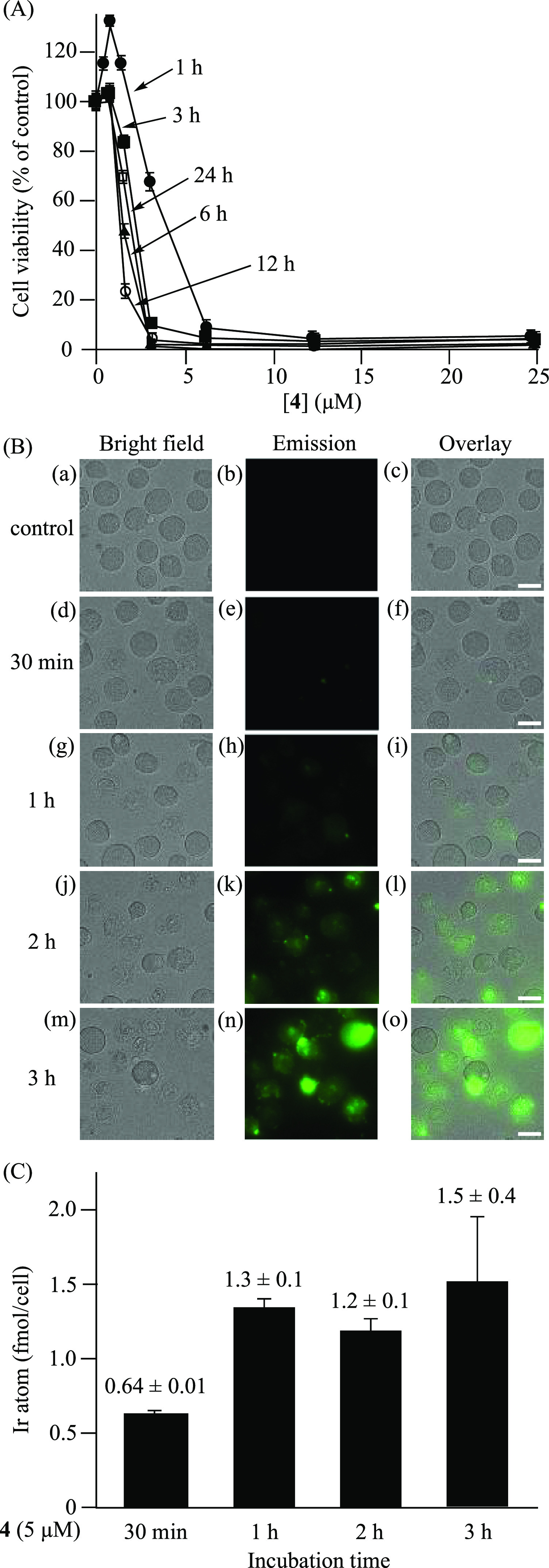
(A) MTT assays of Jurkat cells treated with **4** (0–25 μM) in 10% FBS/RPMI medium at 37 °C under 5% CO_2_ for 1 h (●), 3 h (■), 6 h (▲), 12 h (○), and 24 h (□). (B) Microscopic observations (20×) of Jurkat cells treated with **4** (5 μM) at 37 °C under 5% CO_2_ for 0–3 h. (a, d, g, j, and m) Bright field images of Jurkat cells. (b, e, h, k, and n) Emission images of **4**. (c) Overlay image of panels a and b. (f) Overlay image of panels d and e. (i) Overlay image of panels g and h. (l) Overlay image of panels j and k. (o) Overlay image of panels m and n. Excitation at 377 nm and emission at 520 nm were used. The scale bar (white) is 10 μm. (C) Measurement of the intracellular Ir atom in Jurkat cells treated with **4** (5 μM) for 30 min, 1 h, 2 h, and 3 h by ICP-MS.

The cytotoxicity of **4** against Jurkat cells was evaluated by conducting MTT assays [MTT = 3-(4,5-dimethyl-2-thiazolyl)-2,5-diphenyl-2*H*-tetrazolium bromide] as a function of time and by microscopic observations to determine the appropriate conditions for mechanistic studies of paraptosis (Figure 1). Jurkat cells were incubated with **4** (0–25 μM) in 10% FBS (fetal bovine serum)/RPMI (Roswell Park Memorial Institute) 1640 medium for 1, 3, 6, 12, and 24 h at 37 °C under 5% CO_2_, and their EC_50_ values were determined to be 3.2, 2.0, 1.5, 1.2, and 1.7 μM, respectively (Figure 1A). In microscopic observations, Jurkat cells were treated with **4** (5 μM) for 30 min, 1 h, 2 h, and 3 h, and a strong green emission from **4** was observed in the dead cells in 1–3 h (Figure 1Bg–o).

The time-dependent intracellular uptake of **4** into Jurkat cells was measured by ICP-MS (inductively coupled plasma-mass spectrometry). Jurkat cells were treated with **4** (5 μM) for 30 min, 1 h, 2 h, and 3 h at 37 °C under 5% CO_2_, washed three times with PBS, and lysed with nitric acid at 4 °C overnight. The lysates were diluted with H_2_O, and the samples were analyzed by ICP-MS. As presented in Figure 1C, the intracellular amount of **4** was increased to 0.64 ± 0.01 fmol/cell after incubation for 30 min and to 1.3 ± 0.1 to 1.5 ± 0.4 fmol/cell after 1–3 h, indicating that the emission enhancement of **4** in Jurkat cells after 1 h is correlated with the intracellular uptake of **4**. On the basis of these results, we decided to perform detailed mechanistic studies of paraptosis after incubation with **4** at 5 μM for 1 h.

### Observations of the Mitochondrion-ER Contact Site in Cell Death Induced by **4**

**Figure 2:**
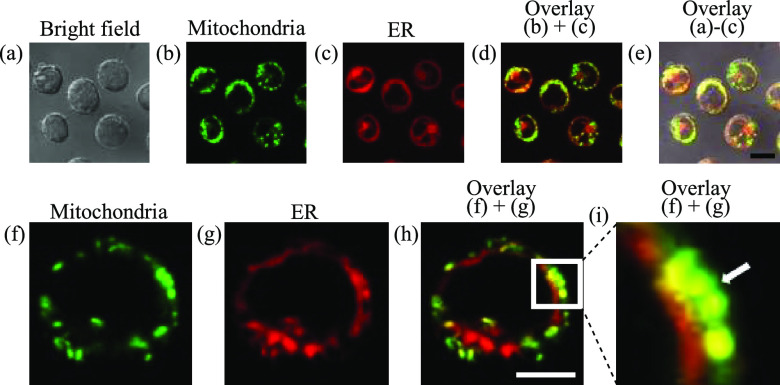
Confocal microscopic images (100×) of Jurkat cells stained with MitoTracker Green and ER-Tracker Red. (a) Bright field image of Jurkat cells. (b and f) Emission images of MitoTracker Green. (c and g) Emission images of ER-Tracker Red. (d) Overlay image of panels b and c. (e) Overlay image of panels a–c. (h) Overlay image of panels f and g. (i) Enlarged image of the white square region in panel h. The tethering site between mitochondria and the ER is indicated by the white arrow. Excitation at 473 nm for MitoTracker Green and 559 nm for ER-Tracker Red were used. Emission from 485 to 545 nm for MitoTracker Green and from 570 to 620 nm for ER-Tracker Red were used. The exposure time: 20 μs/pixel. Scale bars are 10 μm (black) and 5 μm (white).

The physiological connection between mitochondria and the ER is well-known as mitochondria-associated membranes (MAMs), which function to mediate intracellular signaling pathways for inducing apoptosis and autophagy, Ca^2+^ transport, the maintenance of mitochondrial morphology, and the regulation of ER-mitochondrion tethering.^64−74^ Therefore, we observed the relationship between mitochondria and the ER in Jurkat cells by conducting co-staining experiments using specific probes, MitoTracker Green (0.5 μM) for mitochondria and ER-Tracker Red (1 μM) for the ER, by confocal microscopy. As shown in Figure 2, the red emission from ER-Tracker Red was observed at a position close to the green emission from MitoTracker Green and they are partially overlapped (Figure 2f–i), indicating a close contact between mitochondria and the ER.

The transfer of Ca^2+^ from the ER to mitochondria across the MAMs has been extensively studied.^75−84^ It has been established that the inositol 1,4,5-triphosphate receptor (IP_3_R) functions as a Ca^2+^ channel to release Ca^2+^ to MAMs and the cytosol under the control of IP_3_ and Ca^2+^^75^ and that the VDAC (voltage-dependent anion channel) on the outer mitochondrial membrane (OMM) and mitochondrial Ca^2+^ uniporter (MCU) complex on the inner mitochondrial membrane (IMM) mediate the transfer of Ca^2+^ from the ER and cytosol to mitochondria.^76^ The mitochondrial permeability transition pore (mPTP), which is a nonspecific channel located on the IMM, allows ions such as Ca^2+^ and small molecules produced by mitochondrial metabolism to pass through the mitochondrial matrix under the control of mitochondrial Ca^2+^ overload and/or oxidative stress.^77,78^

Some inhibitors of Ca^2+^ channels, including 2-aminophenyl borate (2-APB, an inhibitor of the IP_3_R),^85^ ruthenium red (RuRed, an inhibitor of the MCU complex),^86^ and ER-000444793 (an inhibitor of the mPTP),^87^ have been reported (the structures of these inhibitors are shown in Chart S1). The effect of these inhibitors on the cytotoxicity by **4** and celastrol, which had been reported to function as a paraptosis inducer,^17,18,56−58^ was examined by microscopic observations and MTT assays (Figures S1 and S2). Jurkat cells were incubated in the presence of these inhibitors for 1 h and then treated with **4** or celastrol for 3 or 12 h, respectively. The morphological changes and strong green emission from **4** were then observed in dead cells, indicating the negligible inhibitory effects of 2-APB, ER-000444793, and RuRed on the cell death induced by **4** and celastrol.

### Induction of Paraptosis in Jurkat Cells by CGP37157

**Figure 3:**
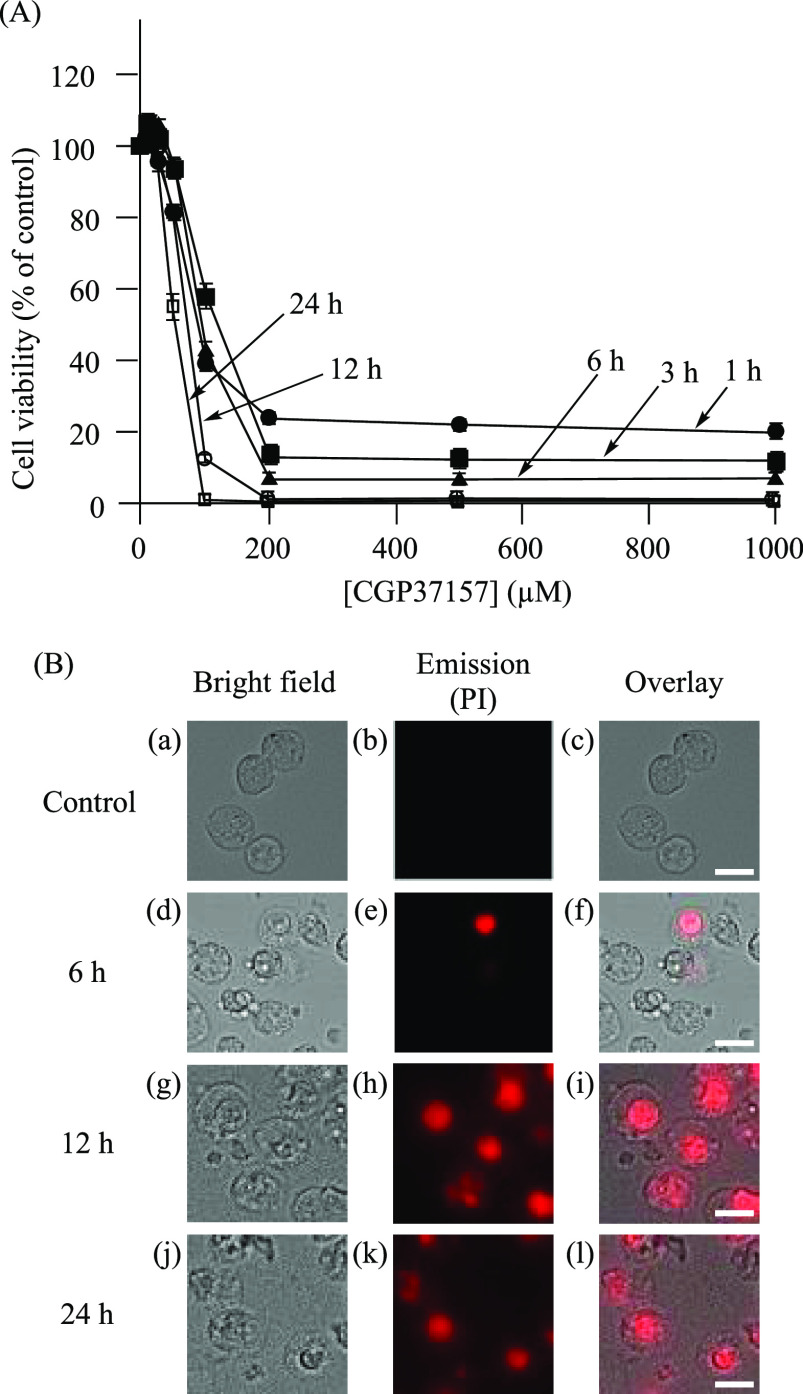
(A) MTT assays of Jurkat cells treated with CGP37157 (0–1000 μM) in 10% FBS/RPMI medium at 37 °C under 5% CO_2_ for 1 h (●), 3 h (■), 6 h (▲), 12 h (○), and 24 h (□). (B) Microscopic observations (40×) of Jurkat cells treated with CGP37157 (100 μM) at 37 °C under 5% CO_2_ for 0–24 h. (a, d, g, and j) Bright field images of Jurkat cells. (b, e, h, and k) Emission images of PI. (c) Overlay image of panels a and b. (f) Overlay image of panels d and e. (i) Overlay image of panels g and h. (l) Overlay image of panels j and k. Excitation at 540 nm and emission at 605 nm were used. The scale bar (white) is 10 μm.

It was reported that an inhibitor of a mitochondrial Na^+^/Ca^2+^ exchanger (mNCX), which functions to export Ca^2+^ from the mitochondrial matrix to the cytosol in exchange with cytosolic Na^+^, affects the mitochondrial Ca^2+^ concentration.^60−63,88−90^ We therefore examined the cytotoxicity of CGP37157 (Chart 2), a typical inhibitor of mNCX,^60,61,88−90^ against Jurkat cells by means of an MTT assay. Jurkat cells were treated with CGP37157 (0–1000 μM) in 10% FBS/RPMI 1640 medium for 1, 3, 6, 12, and 24 h at 37 °C under 5% CO_2_, and the EC_50_ values were determined to be 74 μM for the 12 h incubation and 55 μM for the 24 h incubation (Figure 3A).^91^ For microscopic observations, Jurkat cells were treated with CGP37157 (100 μM) for 0–24 h, and cell death was observed after treatment with CGP37157 for 12–24 h, by staining with propidium iodide (PI) (Figure 3B). Note that the cell viability does not decrease to 0% even at the high concentrations of CGP37157 in Figure 3A, possibly due to its low solubility in water.

**Figure 4:**
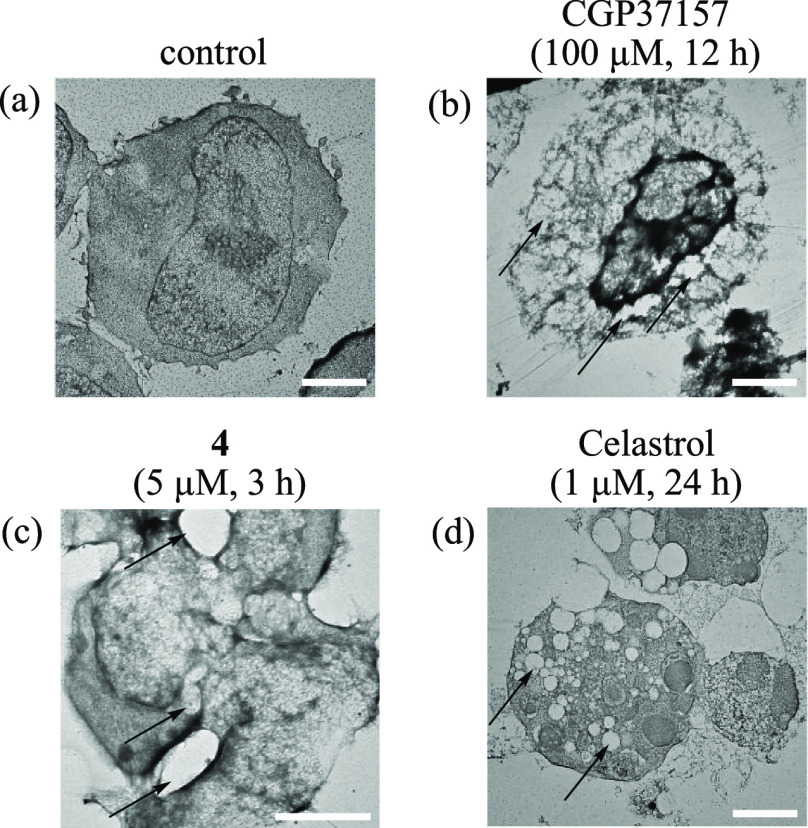
Transmission electron microscopy (TEM) analyses of Jurkat cells treated with (a) no compound, (b) CGP37157 (100 μM, 12 h), (c) **4** (5 μM, 3 h), and (d) celastrol (1 μM, 24 h) at 37 °C under 5% CO_2_. Arrows in panels b–d indicate vacuolization in the cytoplasm induced by CGP37157, **4**, and celastrol, respectively. The white scale bar is 2 μm.

The morphological changes in Jurkat cells induced by CGP37157 (Figure 3B) were similar to those induced by **4** (Figure 1B) and confirmed in detail by TEM (transmission electron microscopy). Jurkat cells were treated with CGP37157 (100 μM) for 12 h, prefixed with glutaraldehyde and postfixed with osmium tetroxide (OsO_4_), and included in Poly 812 resin. The resulting samples were sliced and then observed by TEM. As shown in Figure 4, cytoplasmic vacuolization, a characteristic phenomenon associated with paraptosis, was induced by CGP37157 (Figure 4b), which was similar to that for **4** and celastrol (panels c and d, respectively, of Figure 4). Similar TEM images were reported with respect to paraptosis-inducing natural compounds by some research groups: (i) paraptosis in MDA-MB 435S cells,^17^ HeLa cells,^18^ and Jurkat cells (Figure 4d and in our previous publications)^56−58^ induced by celastrol, (ii) paraptosis in HeLa cells induced by 8-*p*-hydroxybenzoyl tovarol,^23^ and (iii) paraptosis in A2780 cells and SKOV-3 cells induced by morusin.^22^ Therefore, we believe that Jurkat cells undergo vacuolization in the paraptotic processes induced by **4** and CGP37157, as well as celastrol.

We then tested several inhibitors of PCD such as Z-VAD-fmk (a broad caspase inhibitor and an apoptosis inhibitor),^92^ necrostatin-1 (Nec-1, a specific inhibitor of RIPK-1 and a necroptosis inhibitor),^93^ and 3-methyladenine [3-MA, an inhibitor of type III phosphatidylinositol 3-kinases (PI3K) and an autophagy inhibitor]^94^ (the chemical structures of these inhibitors are shown in Chart S2) with respect to the cell death induced by CGP37157. It was found that Z-VAD-fmk weakly inhibited the paraptosis induced by CGP37157, while negligible inhibition by other PCD (necroptosis and autophagy) inhibitors was observed (Figure S4), indicating very weak relationships between the cell death induced by CGP37157 and both necroptosis and autophagy.

**Figure 5:**
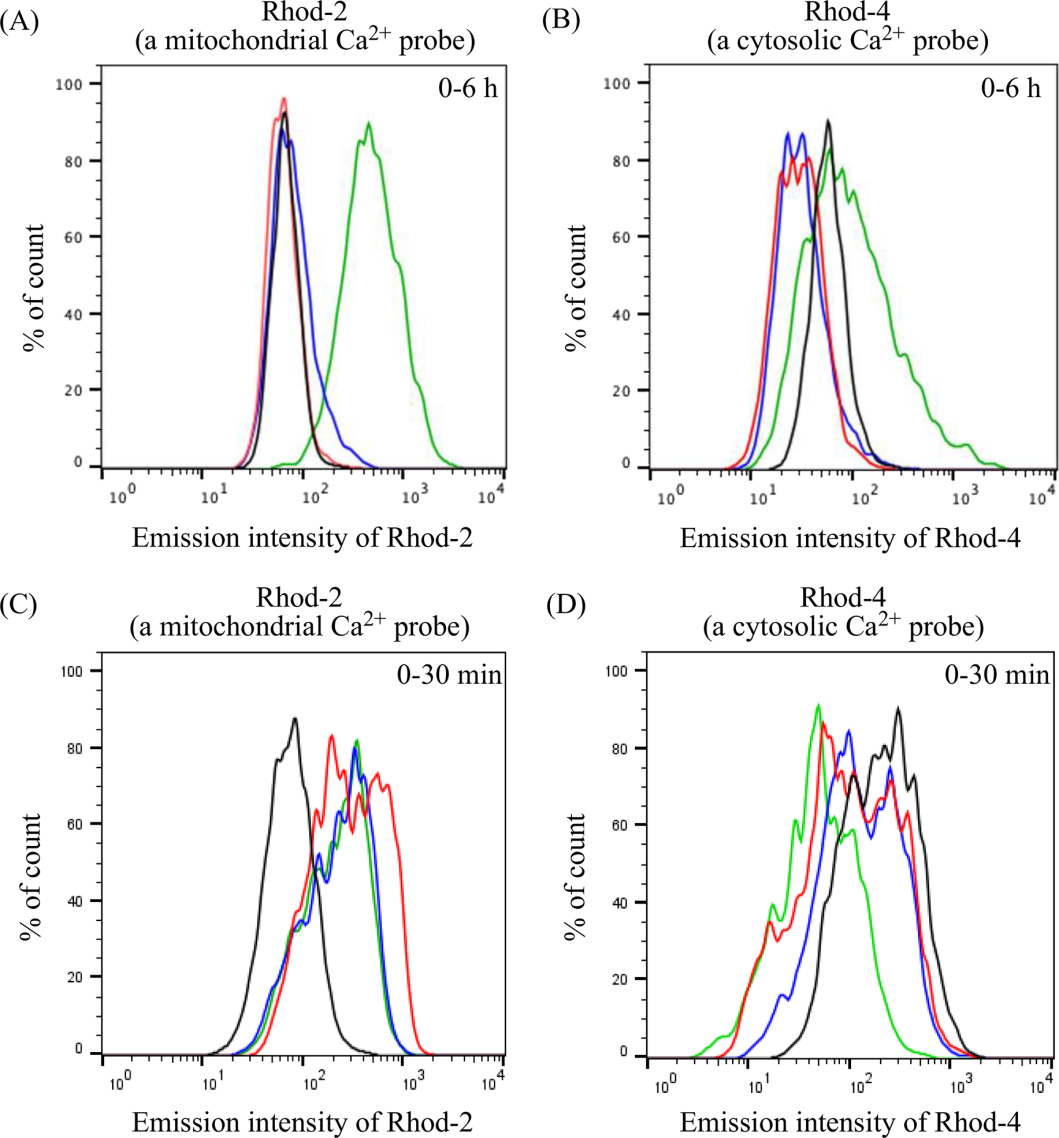
Flow cytometric analysis of Jurkat cells treated with (A and B) CGP37157 (100 μM) and (C and D) **4** (5 μM). Jurkat cells were stained with (A and C) Rhod-2/AM (a mitochondrial Ca^2+^ probe) and (B and D) Rhod-4/AM (a cytosolic Ca^2+^ probe), and then treated with CGP37157 or **4** for a given incubation time. Cytometry profiles at different incubation times for **4** and CGP37157 are shown in different colors: control (black), 1 h (red), 3 h (blue), and 6 h (green) in panels A and B and control (black), 10 min (red), 20 min (blue), and 30 min (green) in panels C and D.

Other characteristic phenomena of paraptosis such as a mitochondrial Ca^2+^ overload and loss of mitochondrial membrane potential (*ΔΨ*_m_) induced by CGP37157 were also examined. Intracellular Ca^2+^ concentrations were measured by flow cytometry using red-emitting Ca^2+^ probes, Rhod-2 (a mitochondrial Ca^2+^ probe) and Rhod-4 (a cytosolic Ca^2+^ probe). Jurkat cells were stained with Rhod-2/AM or Rhod-4/AM (at 5 μM) and then treated with CGP37157 (100 μM) or **4** (5 μM) for a given period of incubation, immediately after which the emission intensity of Rhod-2 and Rhod-4 was measured by flow cytometry. As shown in panels A and C of Figure 5, the emission intensity of Rhod-2 was enhanced 6 h after the treatment with CGP37157 and 10–30 min after the treatment with **4**, indicating the induction of a mitochondrial Ca^2+^ overload by CGP37157 and **4**. On the contrary, a small emission enhancement of Rhod-4 was observed in the cytosol in the presence of CGP37157 and **4** (Figure 5B,D), suggesting that CGP37157 and **4** induce the direct transfer of Ca^2+^ into mitochondria, possibly from the ER, an intracellular Ca^2+^-storing organelle, as proposed in our previous studies.^56−58^

**Figure 6:**
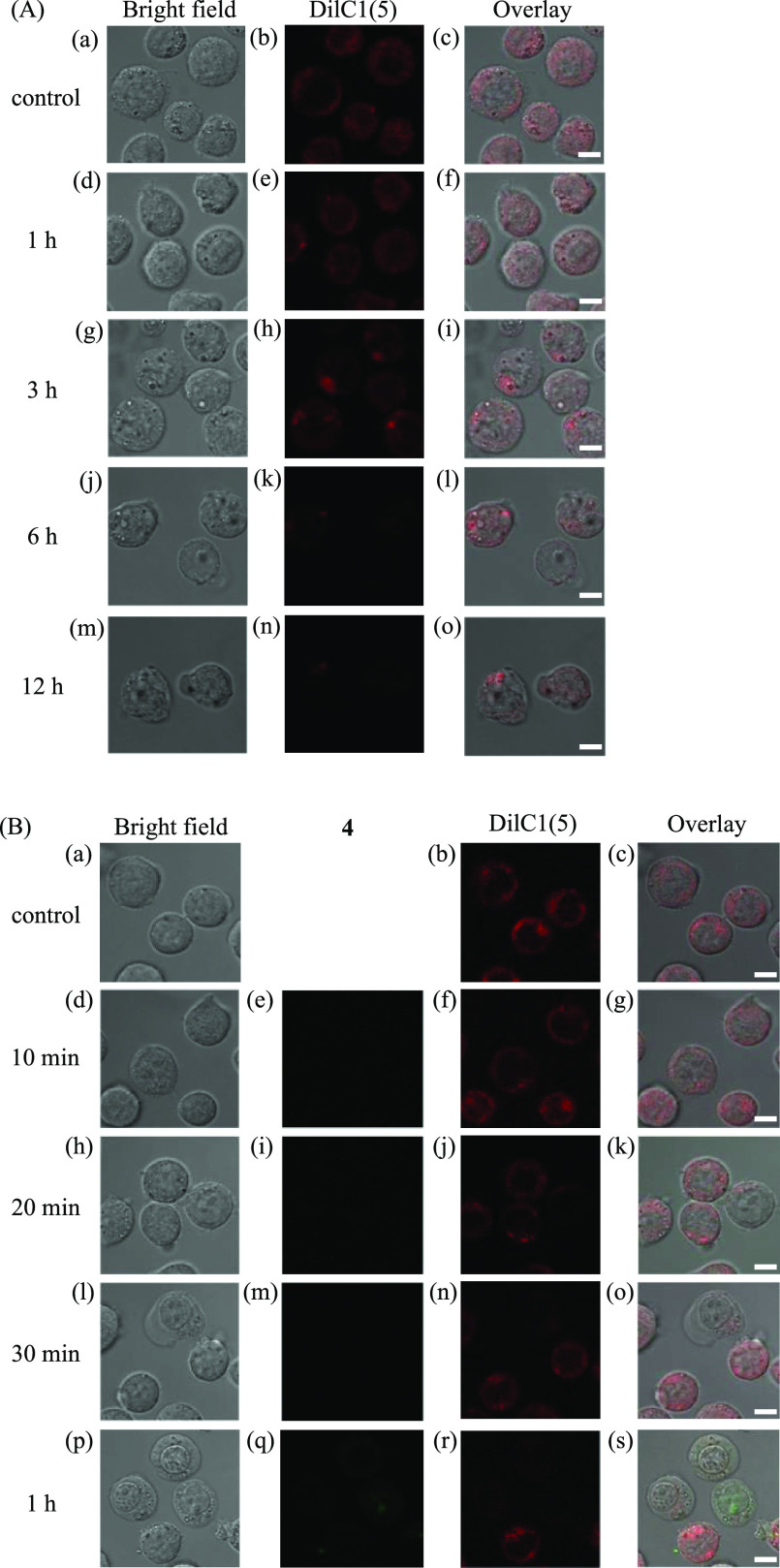
Confocal microscopic observations (100×) of Jurkat cells treated with (A) CGP37157 (100 μM) and (B) **4** (5 μM) in 10% FBS/RPMI medium for 0–12 and 0–1 h, respectively, at 37 °C under 5% CO_2_. The mitochondrial membrane potential (*ΔΨ*_m_) was detected by staining with DilC1(5) (0.5 μM) for 30 min at 37 °C under 5% CO_2_. (A) (a, d, g, j, and m) Bright field images of Jurkat cells. (b, e, h, k, and n) Emission images of DilC1(5). (c) Overlay image of panels a and b. (f) Overlay image of panels d and e. (i) Overlay image of panels g and h. (l) Overlay image of panels j and k. (o) Overlay image of panels m and n. (B) (a, d, h, l, and p) Bright field images of Jurkat cells. (e, i, m, and q) Emission images of **4**. (b, f, j, n, and r) Emission images of DilC1(5). (c) Overlay image of panels a and b. (g) Overlay image of panels d–f. (k) Overlay image of panels h–j. (o) Overlay image of panels l–n. (s) Overlay image of panels p–r. Excitation at 405 nm and emission from 470 to 520 nm for **4** were used. Excitation at 635 nm and emission from 650 to 750 nm for DilC1(5) were used. The exposure time was 20 μs/pixel. The scale bar (white) is 5 μm.

**Figure 7:**
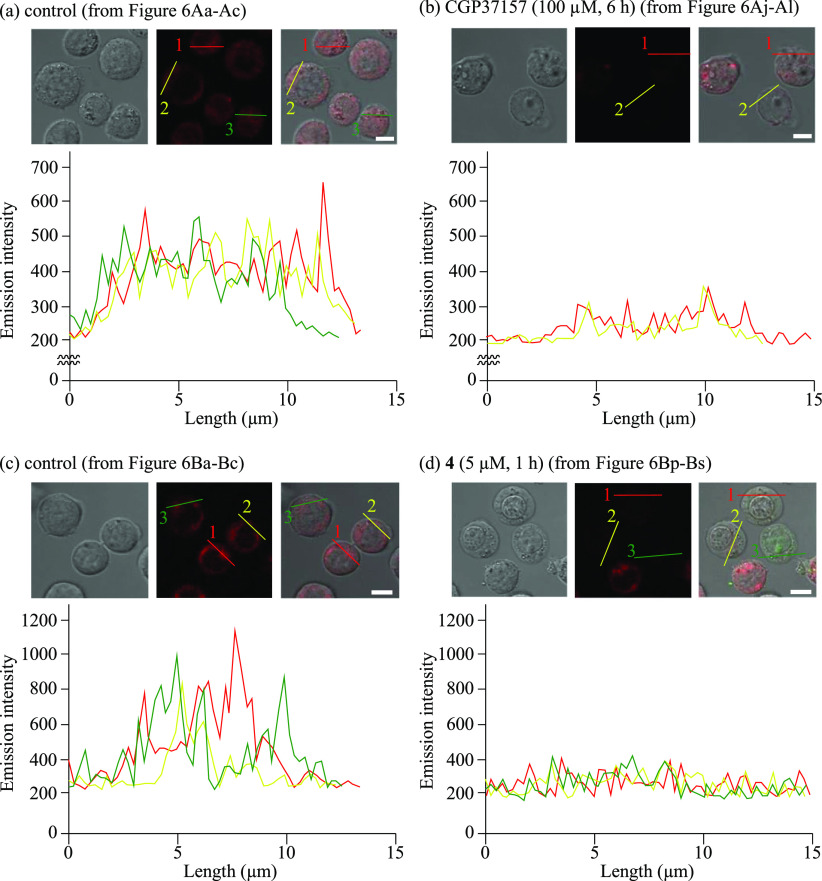
Emission intensity profiles of DilC1(5) obtained from (a) Figure 6Ab, (b) Figure 6Ak, (c) Figure 6Bb, and (d) Figure 6Br. Different colors indicate the intensity profiles of different cells: red for cell 1, yellow for cell 2, and green for cell 3. The scale bar (white) is 10 μm.

The mitochondrial membrane potential (*ΔΨ*_m_) was measured by means of DilC1(5) (1,1′,3,3,3′,3′-hexamethylindodicarbocyanine iodide), the emission intensity of which responds to *ΔΨ*_m_.^95^ Jurkat cells were treated with CGP37157 (100 μM) for 1–12 h, stained with DilC1(5) (5 μM) for 30 min, and observed via confocal microscopy. The red emission of DilC1(5) was quenched after the treatment with CGP37157 for 6 h, as shown in Figure 6A, indicating the decrease in *ΔΨ*_m_. The decrease in *ΔΨ*_m_ with **4** was also observed by confocal microscopy (Figure 6B). Jurkat cells were stained with DilC1(5) and then treated with **4** (5 μM) for 10–30 min, and the decrease in emission intensity was observed 20 min after the treatment with **4**. The change in the emission intensity profiles of DilC1(5) in Figure 6 was analyzed as shown in Figure 7, which clearly shows the loss of *ΔΨ*_m_, triggered by CGP37157 (Figure 7a vs Figure 7b) and **4** (Figure 7c vs Figure 7d).

### Induction of Membrane Fusion between Mitochondria and the ER by **4** and CGP37157

**Figure 8:**
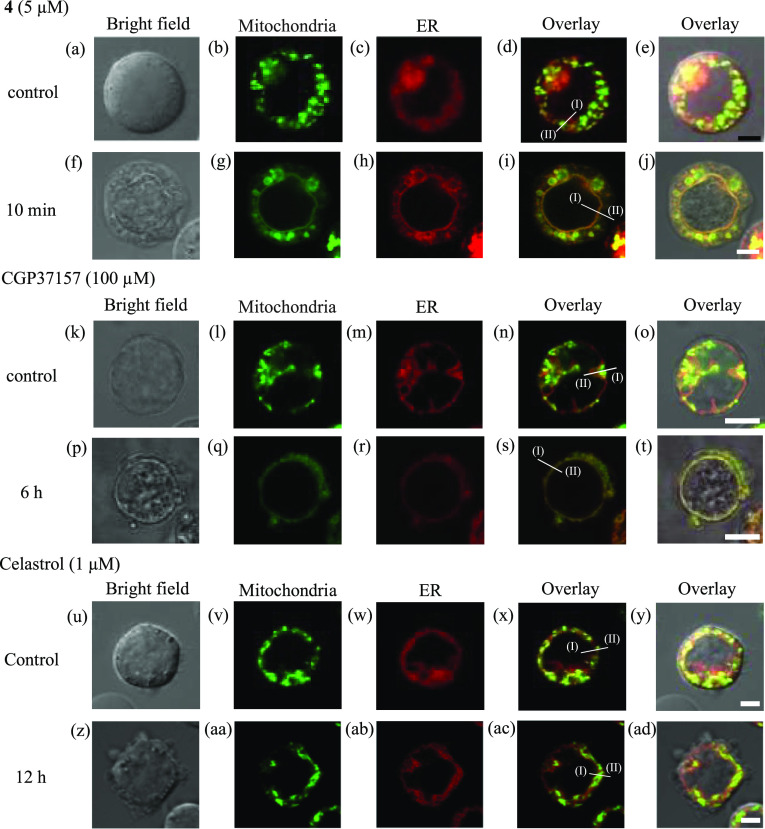
Confocal microscopic images (100×) of Jurkat cells stained with MitoTracker Green (0.5 μM, 1 h) and ER-Tracker Red (1 μM, 1 h) in 10% FBS/RPMI medium and treated with **4** (5 μM, 10 min), CGP37157 (100 μM, 6 h), and celastrol (1 μM, 12 h) in the medium at 37 °C under 5% CO_2_. (a, f, k, p, u, and z) Bright field images of Jurkat cells. (b, g, l, q, v, and aa) Emission images of MitoTracker Green. (c, h, m, r, w, and ab) Emission images of ER-Tracker Red. (d) Overlay image of panels b and c. (e) Overlay image of panels a–c. (i) Overlay image of panels g and h. (j) Overlay image of panels f–h. (n) Overlay image of panels l and m. (o) Overlay image of panels k–m. (s) Overlay image of panels q and r. (t) Overlay image of panels p–r. (x) Overlay image of panels v and w. (y) Overlay image of panels u–w. (ac) Overlay image of panels aa and ab. (ad) Overlay image of panels z–ab. Excitation at 473 nm and emission from 485 to 545 nm were used for MitoTracker Green. Excitation at 559 nm and emission from 570 to 620 nm were used for ER-Tracker Red. The exposure time was 20 μs/pixel. Scale bars are 10 μm (black) and 2 μm (white). The emission intensity profiles of MitoTracker Green and ER-Tracker Red from the point I to II in panels d, i, n, s, x, and ac are shown in Figure 9.

**Figure 9:**
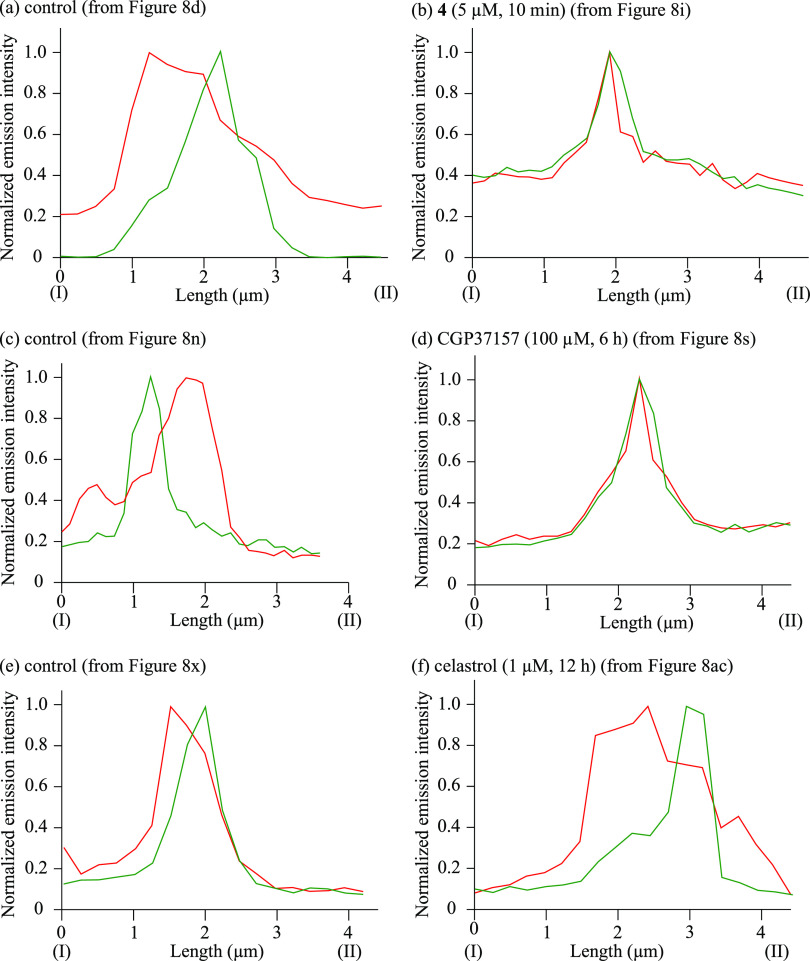
Emission intensity profiles of MitoTracker Green (green) and ER-Tracker Red (red) from point I to II in panels d, i, n, s, x, and ac of Figure 8.

We hypothesized that **4** and CGP37157 would induce membrane fusion between mitochondria and the ER, thus allowing the direct transfer of Ca^2+^ from the ER to mitochondria. To verify this hypothesis, mitochondria and the ER were stained with both MitoTracker Green and ER-Tracker Red in the presence of **4**, CGP37157, and celastrol (Figure 8). Jurkat cells were stained with MitoTracker Green (0.5 μM) and ER-Tracker Red (1 μM) and then treated with **4** (5 μM) for 10–30 min (time-dependent microscopic images are presented in Figure S5). It should be noted that the emission from **4** in Jurkat cells was very weak (excitation at 473 nm for MitoTracker Green, not at 377 nm for the excitation of **4**) during a 1 h incubation (Figure S6), indicating that green emission in Figure 8 is mainly from MitoTracker Green. With regard to CGP37157 and celastrol, Jurkat cells were stained with MitoTracker Green (0.5 μM) and ER-Tracker Red (1 μM) and then treated with CGP37157 (100 μM) and celastrol (1 μM) for 1–24 h (Figure 8 and Figure S5). The emission intensity profiles of these intracellular probes indicated by white lines in panels d, i, n, s, x, and ac of Figure 8 are discussed in Figure 9 and Figure S7. In panels a–e, k–o, and u–y of Figure 8, mitochondria had fragmentated and spherical morphologies in the absence of **4**, CGP37157, and celastrol, respectively, indicating the “fission state” of mitochondria. The mitochondrial structure was changed from a fragmentated feature to a tubule structure that was distributed around the nucleus after the treatment with **4** (5 μM, 10 min) and CGP37157 (100 μM, 6 h) (Figure 8g,q), indicating the development of the “fusion state” of mitochondria. In addition, the green emissions from MitoTracker Green and the red emissions from ER-Tracker Red were extensively overlapped, indicating membrane fusion between mitochondria and the ER (Figure 8f–j,p–t). On the contrary, a fragmentated mitochondrial structure was still observed 12 h after the treatment with celastrol, and the overlap of the emission from MitoTracker Green and ER-Tracker Red was negligible (Figure 8z–ad).

The emission intensity profiles of MitoTracker Green and ER-Tracker Red in Figure 8 are compared, and a detailed analysis of the distribution of mitochondria and the ER was conducted. In Figure 9 and Figure S7, the emission intensity profiles of MitoTracker Green and ER-Tracker Red from point I to II in panels d, i, n, s, x, and ac of Figure 8 and from point III to IV and from point V to VI in Figure S7 are shown as green and red curves, respectively, in each figure. It was observed that the areas of mitochondria and the ER partially overlapped before addition of **4** (Figure 9a and Figure S7a), CGP37157 (Figure 9c and Figure S7c), and celastrol (Figure 9e and Figure S7e). Interestingly, extensive overlap of MitoTracker Green and ER-Tracker Red was observed after the treatment with **4** (Figure 9b and Figure S7b) and CGP37157 (Figure 9d and Figure S7d). In contrast, the distribution (and weak overlap) of mitochondria and the ER was negligibly changed after the treatment with celastrol (1 μM, 24 h), as shown in Figure 9f and Figure S7f. These results strongly suggest that **4** and CGP37157 induce membrane fusion between mitochondria and the ER and that celastrol does not.

### Measurements of Intracellular Guanosine Triphosphatases (GTPases) Related to Mitochondria-ER Membrane Fusion

We next investigated mitochondrial fusion and fission cycle, which is mediated by intracellular guanosine triphosphatases (GTPases) and controls various aspects of mitochondrial function such as energy metabolism and Ca^2+^ homeostasis.^96−100^ The fusion of the mitochondrial membrane includes the outer membrane fusion, which is induced by mitofusin 1 (MFN1) and mitofusin 2 (MFN2), and the inner membrane fusion induced by optic atrophy 1 (OPA1).^99,100^ It was reported that MFN1 and MFN2 are localized on the OMM and ER membrane and exist as homo- or heterodimers that function to induce the membrane fusion of mitochondria.^101,102^ It has also been reported that these GTPases function to construct mitochondria-ER tethering sites for Ca^2+^ transport.^103−106^ The fission of the mitochondrial membrane is mediated by dynamin-related protein 1 (DRP1), which is assembled on the OMM.^107−110^

**Figure 10:**
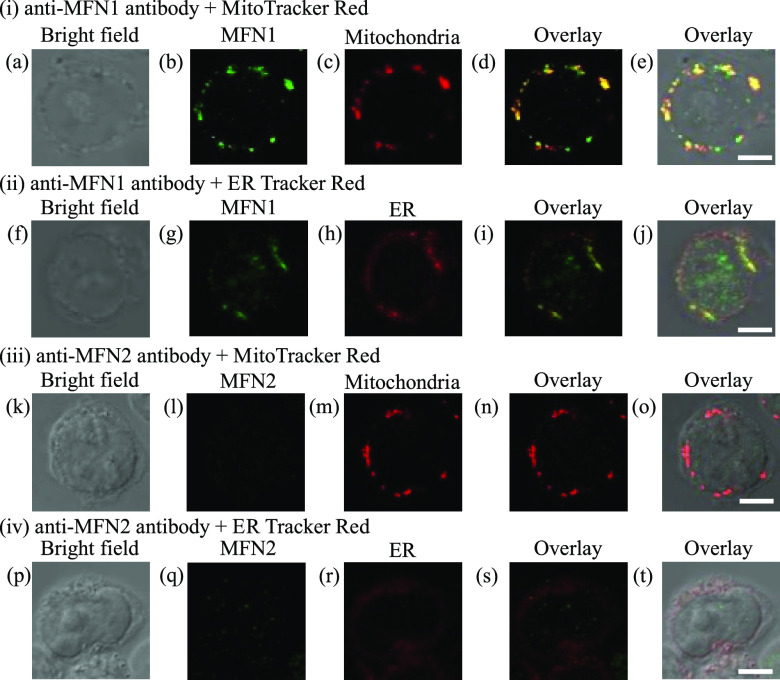
Confocal microscopic images (100×) of Jurkat cells stained with the anti-MFN1 antibody, the anti-MFN2 antibody (1:100 dilution), MitoTracker Red (0.5 μM), and ER-Tracker Red (1 μM). (a, f, k, and p) Bright field images of Jurkat cells. (b and g) Emission images of MFN1. (l and q) Emission images of MFN2. (c and m) Emission images of mitochondria. (h and r) Emission images of the ER. (d) Overlay image of panels b and c. (e) Overlay image of panels a–c. (i) Overlay image of panels g and h. (j) Overlay image of panels f–h. (n) Overlay image of panels l and m. (o) Overlay image of panels k–m. (s) Overlay image of panels q and r. (t) Overlay image of panels p–r. Excitation at 559 nm and emission from 570 to 620 nm were used for MitoTracker Red and ER-Tracker Red. Excitation at 635 nm and emission from 650 to 750 nm were used for the secondary antibody. The exposure time was 20 μs/pixel. The scale bar (white) is 5 μm.

Considering the partial overlaps of MitoTracker Green and ER-Tracker Red in Jurkat cells (Figure 2) and the membrane fusion between mitochondria and the ER in the presence of **4** and CGP37157 (Figures 8 and 9), we carried out cross co-staining experiments with MitoTracker Red and ER-Tracker Red with anti-MFN1 and anti-MFN2 antibodies. In these experiments, Jurkat cells were first stained with MitoTracker Red or ER-Tracker Red, fixed, permeabilized, and blocked, after which the cells were treated with anti-MFN1 or -MFN2 primary antibodies, and an Alexa Fluor 647-conjugated secondary antibody, and then observed by confocal microscopy. Figure 10 shows the results of these cross co-stainings: (i) anti-MFN1 antibody and MitoTracker Red (Figure 10a–e), (ii) anti-MFN1 antibody and ER-Tracker Red (Figure 10f–j), (iii) anti-MFN2 antibody and MitoTracker Red (Figure 10k–o), and (iv) anti-MFN2 antibody and ER-Tracker Red (Figure 10p–t). The green emission from MFN1 and the red emission from MitoTracker Red and ER-Tracker Red extensively overlapped, indicating the co-localization of MFN1 and mitochondria and the ER (Figure 10d,i). On the contrary, the emission from MFN2 in panels l and q of Figure 10 was weak, indicating low expression levels of MFN2 in Jurkat cells.

**Figure 11:**
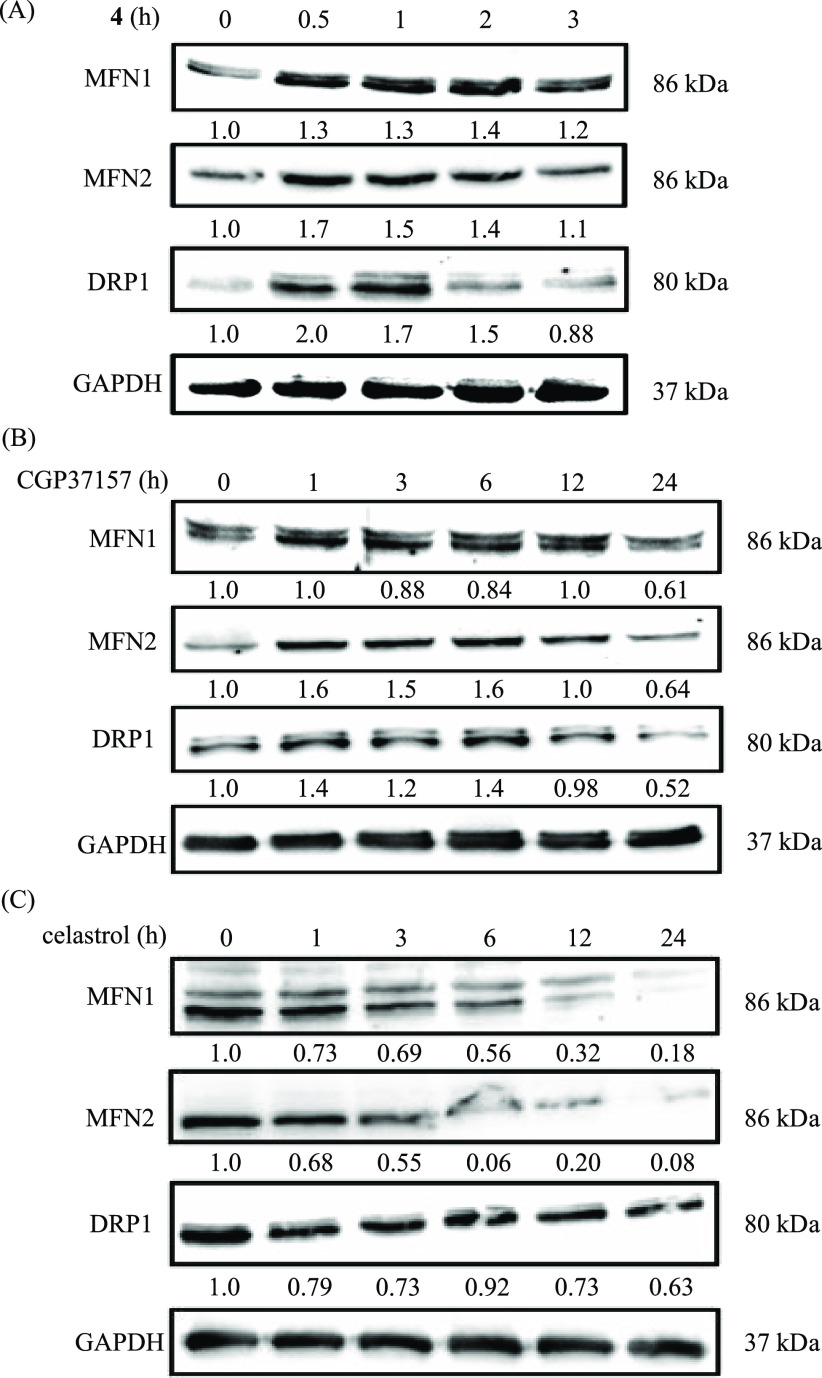
Western blot analyses of mitofusin 1 (MFN1), mitofusin 2 (MFN2), and dynamin-related protein 1 (DRP1) in Jurkat cells (A) treated with **4** (5 μM) for 0–3 h, (B) treated with CGP37157 (100 μM) for 0–24 h, and (C) treated with celastrol (1 μM) for 0–24 h at 37 °C under 5% CO_2_. The intensity of each band was compared on the basis of the condition in the absence of **4**, celastrol, and CGP37157, and the values are shown at the ends of the bands.

The changes in expression levels of MFNs and DRP1 in Jurkat cells by the treatment with **4**, CGP37157, and celastrol were evaluated by Western blot analyses, and the results are shown in Figure 11 and Figure S8. Jurkat cells were incubated in the presence of **4** (5 μM) for 0–3 h, celastrol (1 μM) for 0–24 h, and CGP37157 (100 μM) for 0–24 h [for 30 min with **4** (0–20 μM), 24 h with celastrol (0–10 μM), and 12 h with CGP37157 (0–100 μM)], and the target proteins were extracted and analyzed by Western blot analyses. The expression levels of MFN1 and MFN2 were weakly changed, and DRP1 was somewhat upregulated by **4** at 5 μM for 30 min to 1 h (Figure 11A) and CGP37157 at 100 μM for 1–6 h (Figure 11B), and at its increasing concentration of **4** (after incubation for 1 h) and CGP37157 (after incubation for 12 h) (panels A and B, respectively, of Figure S8). In contrast, celastrol decreased the expression levels of these proteins in a time- and concentration-dependent manner (Figure 11C and Figure S8C).

It has been reported that intracellular GTPases are related to signal transduction, endocytosis, and protein biosynthesis for cell differentiation and proliferation^111,112^ and have also been proposed to be target proteins for cancer therapy.^112^ Therefore, we tested the effects of GTPase inhibitors on the cell death induced by **4** and CGP37157 (the enhancement of expression level of DRP1 by **4** was observed in Figure 11A). It was reported that dynasore inhibits the GTPase activities of dynamin 1/2 and DRP1^113^ and that Mdivi-1 (mitochondrial division inhibitor 1) selectively inhibits DRP1.^114^ CID1067700 has been reported to be a common inhibitor of GTPase, especially the Ras superfamily, which is important in the cell progression through the cell cycle, regulation of cell morphology, and cell invasion and migration^115,116^ (the structures of these inhibitors are shown in Chart S3). Jurkat cells were incubated in the presence of these inhibitors for 1 h, treated with **4** (5 μM, 1 or 3 h) or CGP37157 (100 μM, 12 h), and subjected to MTT assays. As shown in Figure S9, however, these GTPase inhibitors have a negligible effect on the cytotoxicity by **4** and CGP37157, indicating that the mechanism of **4**- and CGP37157-induced paraptosis is unlikely associated with dynamin-related endocytosis, DRP1-mediated mitochondrial fission, and the Ras signaling pathway.

The roles of MFNs in the cell death induced by **4** and CGP37157 were examined by using small interfering RNA (siRNA) for MFN1 and MFN2 to knock down (KD) these proteins. The siRNAs for MFN1 (siRNA_MFN1_) and MFN2 (siRNA_MFN2_) (5 or 10 nM) were added to Jurkat cells, and then the cells were incubated for 48 or 72 h at 37 °C under 5% CO_2_ to produce MFN1- and/or MFN2-KD Jurkat cells. Western blot analyses suggested that MFN1 and MFN2 were knocked down by the corresponding siRNA (10 nM, 48 h) by 45% and 80%, respectively (Figure S10A). The cytotoxicity of **4** and CGP37157 against MFN1- and/or MFN2-KD Jurkat cells was evaluated by MTT assays. The results presented in panels B and C of Figure S10 indicate that the knockdown of MFNs weakly inhibited the cell death induced by **4** and CGP37157. The effect of a negative control siRNA (NCsiRNA) on the expression levels of MFNs and the cell viability of Jurkat cells was negligible, implying that MFNs are somewhat related to the paraptosis induced by **4** and CGP37157. It should be noted that the treatment with siRNA at 5 nM for 72 h was toxic against Jurkat cells in the presence of **4** and CGP37157, although the efficiency of knockdown of these proteins by siRNA could be improved by increasing the incubation time, as shown in Figure S11.

The cytotoxicity of celastrol against MFN1- or MFN2-KD Jurkat cells was also evaluated by MTT assays. As shown in Figure S12, a negligible effect of knockdown of MFNs on the cell death induced by celastrol was observed.

### Plausible Mechanism for the Paraptosis That Is Induced by **4** and CGP37157

**Chart 3:**
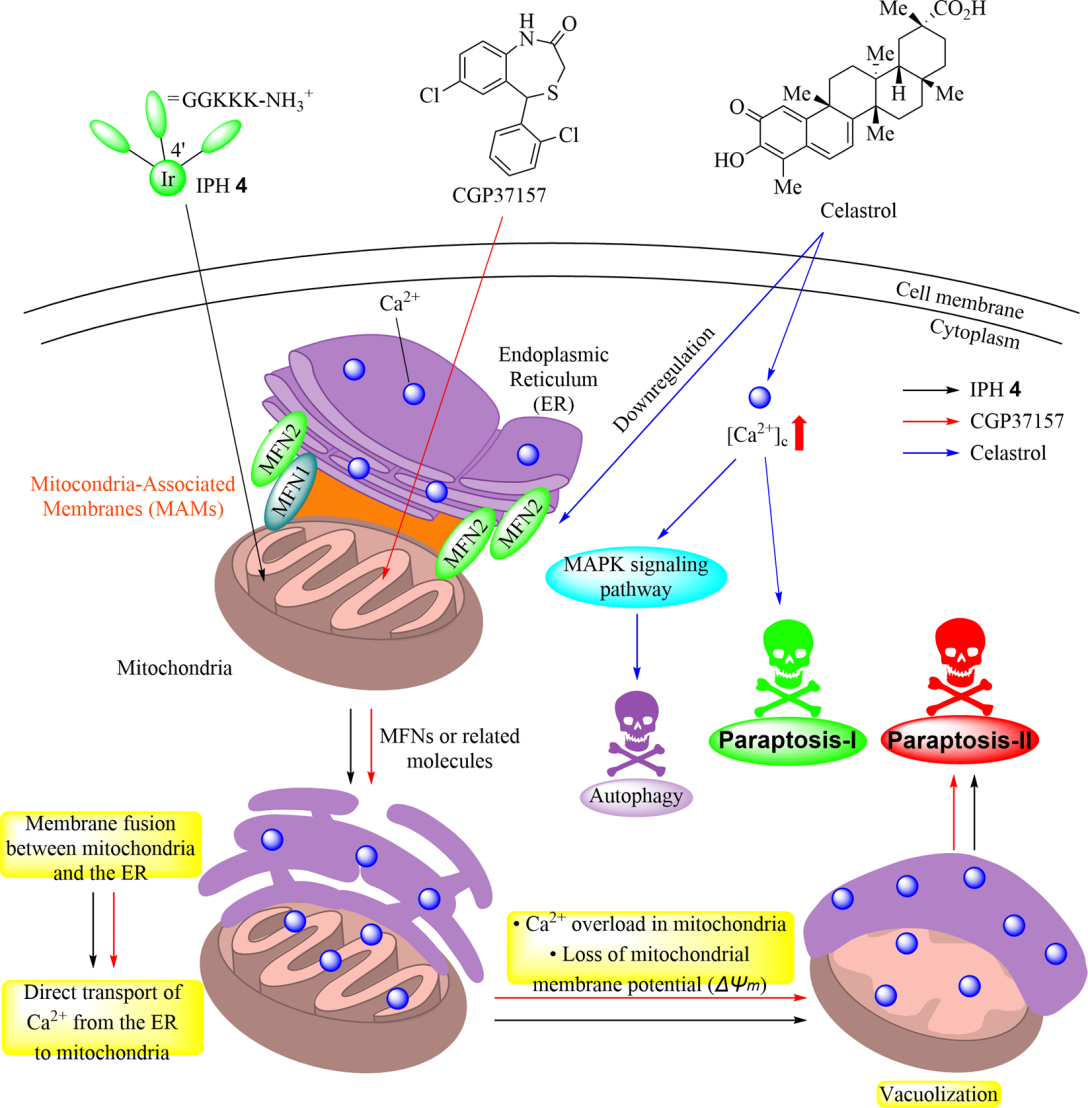
Proposed Schemes for Two Types of Paraptotic Cell Death, Paraptosis I Induced by Celastrol and Paraptosis II Induced by IPH **4** and CGP37157

On the basis of the aforementioned results, proposed plausible mechanisms for the development of paraptosis in Jurkat cells induced by IPH **4**, CGP37157, and celastrol are shown in Chart 3. In Chart 3, black, red, and blue arrows show the plausible mechanistic pathways of paraptosis induced by **4**, CGP37157, and celastrol, respectively.

(1) The Ca^2+^ channels on the membrane of the ER and mitochondria such as IP_3_R, MCU, and mPTP are not major factors in the transport of Ca^2+^ from the ER to mitochondria that is induced by **4**, as confirmed by MTT assays using specific channel inhibitors (Figure S1).

(2) Close contact between mitochondria and the ER via mitochondria-associated membranes (MAMs) was observed in Jurkat cells before the addition of IPHs (Figures 2, 8, and 9). We suggested that **4** induces (i) membrane fusion (or tethering) between mitochondria and the ER via MFNs and related molecules (paraptosis induced by **4** was partially suppressed by the knockdown of MFNs), (ii) direct transport of Ca^2+^ from the ER to mitochondria, and (iii) a decrease in the mitochondrial membrane potential (*ΔΨ*_m_), resulting in paraptosis in Jurkat cells (Figures 4–9 and Figures S5, S7, and S10). We assume that the induction of the membrane fusion of mitochondria and the ER by artificial compounds is one of the most important findings in this work.

(3) We found that CGP37157, a mNCX inhibitor, induces paraptosis in Jurkat cells after treatment for 12–24 h (EC_50_ value of 55 μM for 24 h), as confirmed by microscopic and TEM observations (Figures 3 and 4). We revealed that CGP37157 also induces membrane fusion between mitochondria and the ER for direct Ca^2+^ transport and a decrease in *ΔΨ*_m_ (Figures 5–9 and Figures S5 and S7). This scenario is supported by the results showing that the knockdown of MFNs partially restored the cell viability of Jurkat cells (Figure S10).

(4) We observed that **4** and CGP37157 weakly change the expression levels of dynamine-related protein 1 (DRP1), a mitochondrial membrane fission protein (Figure 11 and Figure S8). A negligible effect of DRP1 inhibitors on paraptosis was observed, indicating that DRP1 is scarcely related to **4**- and CGP37157-induced paraptosis (Figures S9).

(5) In the presence of celastrol, which had been reported to be a paraptosis inducer,^17,18,56−58^ the expression levels of MFNs are suppressed and membrane fusion between mitochondria and the ER was negligible (Figures 8, 9, and 11 and Figures S5, S7, and S8). We previously reported that celastrol increases Ca^2+^ concentrations in the cytoplasm rather than in mitochondria for the induction of paraptosis and activates the mitogen-activated protein kinase (MAPK) signaling pathway, resulting in autophagy.^56−58^ These data imply that different signaling pathways are involved in paraptosis induced by different drugs.

(6) In our previous study, we reported that IPHs such as **2–4** are localized on mitochondria during the early stages of the cell death process.^52,56−58^ These data, considering that CGP37157 is a mNCX inhibitor,^60,61,88−90^ strongly suggest that the main target organelle of these compounds is the mitochondria. On the contrary, several target proteins of celastrol have been identified,^117−120^ such as a heat-shock protein 90 (Hsp90)-cell division cycle 37 (Cdc37) complex that controls protein folding^118^ and IκB kinase (IKK) that regulates gene transcription through NF-κB activation.^119^ In addition, it has been proposed that a quinone methide moiety of celastrol functions as an acceptor of Michael addition (1,4-addition) reactions with the thiol residue of the target proteins.^8,119,120^ Although the relationship between these target proteins of celastrol and paraptotic mechanisms in Jurkat cells remains unclear, it is very likely that these differences result in the different mechanisms in paraptosis induced by **4** and CGP37157 and in paraptosis induced by celastrol.

These findings allow us to suggest that paraptosis can be classified into at least two types. The first is a known type of paraptosis (termed paraptosis I herein) that is induced by celastrol and negligibly involves membrane fusion between mitochondria and the ER. The second type of paraptosis (termed paraptosis II herein) induced by IPHs such as **2–4** and CGP37157 is associated with membrane fusion between mitochondria and the ER.

## Conclusions

In summary, we report on the results of a series of more detailed mechanistic studies of paraptotic cell death that is induced by Ir complex-peptide hybrids (IPHs) that possess basic (cationic) peptides, focusing on the direct transport of Ca^2+^ from the ER to mitochondria. The findings suggest that IPH **4** induces membrane fusion (or tethering) between the ER and mitochondria. We also found that CGP37157, an inhibitor of a mitochondrial Na^+^/Ca^2+^ exchanger (mNCX), induces paraptosis in Jurkat cells via intracellular pathways similar to those induced by **4**. Importantly, the membrane fusion of the ER and mitochondria by these two compounds would lead to the direct transport of Ca^2+^ from the ER to mitochondria. In contrast, celastrol, which had been known as a naturally occurring paraptosis inducer, negligibly has such a function. To the best our knowledge, this is the first example to show the structural fusion of mitochondria with the ER by artificial molecules and the direct transfer of Ca^2+^ from the ER to mitochondria to stimulate intracellular pathways for the induction of programmed cell death.

The results obtained in this work indicate that paraptosis should be classified into two types. The first is a known type of paraptosis induced by celastrol, which is termed paraptosis I herein, which involves Ca^2+^ overload in the cytoplasm and hardly involves membrane fusion between mitochondria and the ER. The second type is a new class of paraptosis induced by **4** and CGP37157 and is termed paraptosis II in this work, which involves mitochondria–ER membrane fusion and subsequent mitochondrial Ca^2+^ overload. Because the structures of IPHs and CGP37157 are so different, we do not exclude the possibility that these two molecules activate different target molecules to stimulate intracellular signaling pathways involved in paraptosis II.

The findings reported in this study provide useful information not only for mechanistic studies of PCD such as paraptosis but also for the design and synthesis of PCD inducers in cancer cells in the future. The design and synthesis of IPHs and other types of peptide hybrids that possess higher anticancer activity and more detailed mechanistic studies are now underway.

## Experimental Section

### General Information

All reagents and solvents were of the highest commercial quality and used without further purification, unless otherwise noted. All aqueous solutions were prepared using deionized water. MitoTracker Green, MitoTracker Red, ER-Tracker Red, and Opti-modified Eagle’s medium (Opti-MEM) were purchased from Invitrogen. 3-(4,5-Dimethyl-2-thiazolyl)-2,5-diphenyl-2*H*-tetrazolium bromide (MTT), Rhod-2/AM, and Rhod-4/AM were purchased from Dojindo. Z-VAD-fmk was purchased from the Peptide Institute, and necrostatin-1 (Nec-1) was purchased from Enzo Life Science. Propidium iodide (PI), 3-methyladenine (3-MA), potassium chloride, and bovine serum albumin (BSA) were purchased from Fujifilm Wako Chemicals. CGP37157 and DilC1(5) (1,1′,3,3,3′,3′-hexamethylindodicarbocyanine iodide) were purchased from Sigma-Aldrich. Roswell Park Memorial Institute (RPMI) 1640 medium, minimum essential medium (MEM), Dulbecco’s modified Eagle’s medium (DMEM), phosphate-buffered saline (PBS), Tween 20, a 40% (w/v) acrylamide/bisacrylamide solution, tris(hydroxymethyl)aminomethane (Tris), sodium dodecyl sulfate (SDS), ammonium persulfate (APS), glycine, sodium chloride, and RuRed were purchased from Nacalai tesque. Dynasore and Mdivi-1 were purchased from TCI (Tokyo Chemical Industry). Celastrol and 2-aminoethoxydiphenylborate (2-APB) were purchased from Cayman Chemical Co. ER-000444793 was purchased from MedChemExpress. CID1067700 was purchased from Calbiochem. The Pierce BCA Protein Assay Kit was purchased from Thermo Fisher Scientific Inc. Fetal bovine serum (FBS) was purchased from Capricorn products Inc. The anti-MFN1 antibody, anti-MFN2 antibodies, anti-DRP1 antibody, and siRNA for MFN1 (siRNA_MFN1_) and MFN2 (siRNA_MFN2_) were purchased from Santa Cruz Biotechnology. The anti-GAPDH, anti-mouse IgG HRP-linked, and anti-rabbit IgG HRP-linked antibodies were purchased from Cell Signaling. The anti-mouse IgG Alexa Fluor 647-linked antibody was purchased from Abcam. INTERFERin was purchased from Pulyplus. Stock solutions of **4** in PBS, CGP37157, and celastrol in DMSO were stored at 0 °C. The results of MTT and BCA assays were confirmed by using a multilabel counter, Wallac 1420 ARVO (PerkinElmer). Fluorescent imaging studies were conducted using fluorescent microscopes (Biorevo, BZ-9000, Keyence, and Fluoview, FV-1000, Olympus). The intracellular uptake of **4** was measured by ICP-MS (NexION300S, PerkinElmer). The results of Western blot analyses were analyzed on the ChemiDoc MP system (Bio-Rad). Flow cytometric analyses were performed by using a flow cytometer (FACSCalibur cytometer, Becton), and data were analyzed on FlowJo software (FlowJo, LCC). TEM images were obtained by using the instrument (H-7650, Hitachi).

### Cell Cultures

Jurkat, HeLa S3, and A549 cells were incubated in RPMI 1640 medium, MEM, and DMEM, respectively, supplemented with 10% heat-inactivated fetal bovine serum (FBS) and 1% penicillin/streptomycin at 37 °C in a humidified 5% CO_2_ incubator.

### MTT Assays

HeLa S3 and A549 cells (2.0 × 10^4^ cells/well) were seeded on a 96-well plate (BD Falcon) in cell culture medium and incubated overnight at 37 °C under 5% CO_2_. Jurkat (2.0 × 10^4^ cells/well), HeLa S3, and A549 cells were treated with **4** (0–25 μM) and CGP37157 (0–100 μM) for 1, 3, 6, 12, and 24 h at 37 °C under 5% CO_2_, after which a MTT solution in PBS (0.5%, 10 μL) was added to each well. After incubation for 4 h at 37 °C under 5% CO_2_, a 10% SDS in 0.01 N HCl aqueous solution (100 μL) was used as a formazan lysis solution and the resulting solutions were incubated overnight under the same conditions, followed by the measurement of the absorbance at 570 nm using a multilabel counter, Wallac 1420 ARVO (PerkinElmer).

### MTT Assays in the Presence of Inhibitors

In a 96-well plate, Jurkat cells (2.0 × 10^4^ cells/well) were incubated in the presence of 2-APB (50 μM), ER-000444793 (10 μM), RuRed (10 μM), Dynasore (10–30 μM), Mdivi-1 (10–30 μM), CID1067700 (1–10 μM), Z-VAD-fmk (30 μM), Nec-1 (30 μM), and 3-MA (5 mM) in 10% FBS RPMI medium (50 μL) for 1 h at 37 °C under 5% CO_2_, and then solutions of **4** (10 μM) and celastrol (2 μM) in 10% FBS/RPMI 1640 medium (50 μL) were added. The final concentrations of **4** and celastrol were 5 and 1 μM, respectively. The resulting solutions were incubated at 37 °C under 5% CO_2_ for 1 and 3 h (**4**) or 24 h (celastrol), after which a 0.5% MTT solution in PBS (10 μL) was added to each well. After incubation for 4 h at 37 °C under 5% CO_2_, a 10% SDS in 0.01 N HCl aqueous solution (100 μL) was used as a formazan lysis solution and the resulting solutions were incubated overnight under the same conditions, followed by the measurement of the absorbance at 570 nm using a multilabel counter, Wallac 1420 ARVO (PerkinElmer).

### Microscopic Observations of Jurkat Cells Treated with **4**, CGP37157, and Celastrol

In a 1.5 mL Eppendorf tube, Jurkat cells (2.0 × 10^5^ cells) were treated with **4** (5 μM), CGP37157 (100 μM), and celastrol (1 μM) in 10% FBS/RPMI 1640 medium (100 μL) at 37 °C under 5% CO_2_ for different periods of incubation time, after which the cells were collected by centrifugation and washed with PBS. For CGP37157 and celastrol, the cells were treated with propidium iodide (100 μM, 100 μL) in PBS for 30 min at 37 °C under 5% CO_2_, washed with PBS, and observed by fluorescence microscopy (Biorevo, BZ-9000, Keyence) using a Greiner CELLview dish (35 mm × 10 mm). Emission images were obtained by using an FF01 filter (excitation at 377 nm and emission at 520 nm) for **4** and a TRITC filter (excitation at 540 nm and emission at 605 nm) for CGP37157 and celastrol.

### Confocal Microscopic Observations of Jurkat Cells Treated with **4**, CGP37157, and Celastrol and Stained with MitoTracker Green and ER-Tracker Red

In a 1.5 mL Eppendorf tube, Jurkat cells (2.0 × 10^5^ cells) were stained with MitoTracker Green (0.5 μM) for 1 h at 37 °C under 5% CO_2_ and then ER-Tracker Red (1 μM) for 1 h at 37 °C under 5% CO_2_ in 10% FBS/RPMI 1640 medium (100 μL). After being washed with PBS, the cells were treated with **4** (5 μM) in 10% FBS/RPMI 1640 medium (100 μL) for 0–30 min at 37 °C under 5% CO_2_. For CGP37157 and celastrol, Jurkat cells were treated with CGP37157 (100 μM) or celastrol (1 μM) in 10% FBS/RPMI 1640 medium (100 μL) for 0–24 h, after which the cells were stained with MitoTracker Green (0.5 μM) for 1 h at 37 °C under 5% CO_2_ and then ER-Tracker Red (1 μM) for 1 h at 37 °C under 5% CO_2_ in PBS. The cells were washed with PBS and observed by confocal microscopy (Fluoview, FV-1000, Olympus) using a Greiner CELLview dish (35 mm × 10 mm). Excitation at 473 nm and emission from 485 to 545 nm were used for MitoTracker Green. Excitation at 559 nm and emission from 570 to 620 nm were used for ER-Tracker Red. The exposure time was 20 μs/pixel.

### Measurement of Intracellular Uptake of **4** in Jurkat Cells Evaluated by ICP-MS

In a 1.5 mL Eppendorf tube, Jurkat cells (1.0 × 10^6^ cells) were treated with **4** (5 μM) for 0–3 h at 37 °C under 5% CO_2_ (*n* = 3). After the cells had been washed three times with PBS, HNO_3_ (60%, 0.5 mL) was added to the cells and the resulting solutions were incubated overnight at 4 °C. After centrifugation (15000 rpm, 4 °C, 10 min), the supernatant was transferred to a 15 mL tube, diluted with H_2_O (4.5 mL), and filtered. The number of iridium atoms was measured by ICP-MS (NexION300S, PerkinElmer).

### Western Blot Analyses

In a 1.5 mL Eppendorf tube, Jurkat cells (1.0 × 10^6^ cells) were incubated in the presence of **4** (5 μM for 0–3 h or 0–20 μM for 30 min), CGP37157 (100 μM for 0–24 h or 0–100 μM for 12 h), and celastrol (0–10 μM for 24 h or 1 μM for 0–24 h) at 37 °C under 5% CO_2_. After the cells had been washed twice with PBS, the proteins were extracted by using RIPA buffer (Nacalai Tesque) and quantified using a Pierce BCA Protein Assay Kit (Thermo Scientific). Proteins (7.5 μg/well) for MFNs and DRP1 and proteins (5.0 μg/well) for GAPDH were used for sodium dodecyl sulfate–polyacrylamide gel electrophoresis (SDS–PAGE) (7.5% for MFNs and DRP1 and 10% for GAPDH) (Bio-Rad). After SDS–PAGE, the proteins were transferred to a polyvinylidene fluoride membrane (Merck Millipore) by using a semi dry blotter (Bio-Rad). The membrane was blocked with Blocking One solution (Nacalai Tesque) for 30 min at room temperature. After being blocked, the membrane was washed three times with 1× TBST and treated with primary antibodies overnight (at a dilution of 1/1000 for MFNs and DRP1 or 1/2000 for GAPDH) in a signal enhancer HIKARI-solution A (Nacalai Tesque). The membrane was washed three times with 1× TBST and treated with the anti-mouse or anti-rabbit IgG HRP-conjugated secondary antibody (at a dilution of 1/4000 for MFNs and DRP1 or 1/10000 for GAPDH) in 1× TBST for 60 min at room temperature. The protein signal was spotted with a Chemi-Lumi One Ultra solution (Nacalai Tesque) using the ChemiDoc MP system (Bio-Rad).

### Transmission Electron Microscopy (TEM) Analyses of Jurkat Cells Treated with **4**, CGP37157, and Celastrol

In a 1.5 mL Eppendorf tube, Jurkat cells (3.0 × 10^6^ cells) were incubated with **4** (5 μM for 3 h), CGP37157 (100 μM for 12 h), and celastrol (1 μM for 24 h) in 10% FBS/RPMI 1640 medium at 37 °C under 5% CO_2_. After centrifugation (2000 rpm, 3 min, 4 °C), the cells were washed twice with PBS, prefixed with glutaraldehyde (2.5%) at 4 °C for 40 min, and then washed twice with PBS. Osmium tetroxide (1%) was used for postfixation, and the solution was incubated at 4 °C for 30 min. The cells were washed with PBS, included in an agarose gel, and dehydrated by using 50–100% and anhydrous EtOH. The cells were embedded into Poly 812 resin (Nisshin EM Co. Ltd.) at 60 °C for 3 days. The resin was sliced with a glass knife (150 nm thickness) on an ultramicrotome (EM UC6, Leica), and the samples were stained with EM stainer (Nisshin EM Co. Ltd.) and observed on TEM instrument (H-7650, Hitachi) with electron irradiation at 100 kV.

### Measurement of Intracellular Ca^2+^ Concentrations of Jurkat Cells Treated with **4** and CGP37157 by Flow Cytometry

In a 1.5 mL Eppendorf tube, Jurkat cells (2.0 × 10^5^ cells) were incubated in the presence of Rhod-2/AM (5 μM, 100 μL) or Rhod-4/AM (5 μM, 100 μL) in 10% FBS/RPMI 1640 medium for 30 min at 37 °C under 5% CO_2_ and then treated with **4** (5 μM, 0–30 min) or CGP37157 (100 μM, 0–6 h) in 10% FBS/RPMI 1640 medium (100 μL) for 30 min at 37 °C under 5% CO_2_. Immediately after the treatment, 10% FBS/RPMI 1640 medium (300 μL) was added to the cells and the samples were analyzed on a flow cytometer (FACSCalibur cytometer, Becton). The data were analyzed on FlowJo software (FlowJo, LCC).

### Measurement of the Mitochondrial Membrane Potential (*ΔΨ*_m_) of Jurkat Cells Treated with **4** and CGP37157

In a 1.5 mL Eppendorf tube, Jurkat cells (2.0 × 10^5^ cells) were stained with DilC1(5) (500 nM, 100 μL) in 10% FBS/RPMI 1640 medium for 30 min at 37 °C under 5% CO_2_, followed by the treatment with **4** (5 μM, 100 μL) in the medium for 0–60 min at 37 °C under 5% CO_2_. For CGP37157, Jurkat cells (2.0 × 10^5^ cells) were treated with CGP37157 (100 μM, 100 μL) in 10% FBS/RPMI 1640 medium for 0–12 h at 37 °C under 5% CO_2_, followed by the treatment with DilC1(5) (500 nM, 100 μL) in PBS. The cells were washed with PBS and observed by confocal microscopy (Fluoview, FV-1000, Olympus) using a Greiner CELLview dish (35 mm × 10 mm). Excitation at 405 nm and emission from 470 to 520 nm were used for **4**. Excitation at 635 nm and emission from 650 to 750 nm were used for DilC1(5). The exposure time was 20 μs/pixel.

### Immunostaining of Mitofusins in Jurkat Cells Stained with MitoTracker Red and ER-Tracker Red

In a 1.5 mL Eppendorf tube, Jurkat cells (1.0 × 10^6^ cells) were stained with MitoTracker Red (0.5 μM, 1 h) or ER-Tracker Red (1 μM, 1 h) in 10% FBS/RPMI 1640 medium at 37 °C under 5% CO_2_. After being washed with PBS, the cells were fixed with 4% paraformaldehyde in PBS (500 μL) for 10 min at 37 °C under 5% CO_2_, washed with PBS, and permeabilized by using 0.1% Tween 20 in PBS (200 μL) for 15 min at room temperature. After the cells had been blocked with 2% BSA in 1× PBST (200 μL) for 1 h at room temperature and washed with 1× PBST, the cells were treated an anti-MFN1 or an anti-MFN2 antibody (1:100 dilution in 1× PBST) at 4 °C overnight. The cells were washed with 1× PBST and treated with anti-mouse IgG H&L (Alexa Fluor 647) (1:100 dilution in 1× PBST) for 1 h at room temperature. After being washed with 1× PBST, the cells were observed by confocal microscopy (Fluoview, FV-1000, Olympus) using a Greiner CELLview dish (35 mm × 10 mm). Excitation at 559 nm and emission from 570 to 620 nm were used for MitoTracker Red and ER-Tracker Red. Excitation at 635 nm and emission from 650 to 750 nm were used for the detection of MFN1 and MFN2. The exposure time was 20 μs/pixel.

### Knockdown (KD) of Mitofusins in Jurkat Cells by Small Interfering RNA (siRNA)

To a solution of transfection reagent (INTERFERin, 5 μL) in OPTI-MEM (0.3 mL) in a 1.5 mL Eppendorf tube was added siRNA (10 μM, 1 or 2 μL) in H_2_O, and the resulting solution was allowed to stand for 15 min at room temperature. The resulting solution was then added to Jurkat cells (2.0 × 10^5^ cells/mL, 1.5 mL) in 10% FBS/RPMI 1640 medium that had been seeded on a 12-well plate and incubated overnight at 37 °C under 5% CO_2_ (the final concentration of siRNA was 5 or 10 nM), and the resulting solution was incubated for 48 or 72 h at 37 °C under 5% CO_2_. The cells were collected by centrifugation (2000 rpm, 3 min, 4 °C), and the expression levels of MFNs were evaluated by Western blot analysis as described above. The cytotoxicity of **4** (3 or 5 μM), CGP37157 (50 or 100 μM), and celastrol (1 μM) against MFNs-KD Jurkat cells was evaluated by MTT assays as described above.

### Statistical Analysis

Statistical analyses of MTT assays were performed by using Graphpad Prism 9 software with the Student’s *t* test. Data are presented as means ± the standard deviation of three independent experiments, and a *P* of <0.05 was considered to indicate a statistically significant difference.

## Supporting Information Available

Effect of 2-APB, ER-000444793, and RuRed on the cell death induced by **4** (Figure S1) and celastrol (Figure S2), MTT assay of HeLa S3 and A549 cells treated with CGP37157 (Figure S3), effect of Z-VAD-fmk, necrostatin-1, and 3-methyladenine on the cell death induced by CGP37157 (Figure S4), confocal microscopic observations of Jurkat cells treated with **4**, CGP37157, and celastrol and stained with MitoTracker Green and ER-Tracker Red (Figure S5), confocal microscopic observations of Jurkat cells treated with **4** (Figure S6), emission intensity profiles of MitoTracker Green and ER-Tracker Red in Figure 8 (Figure S7), Western blot analyses of intracellular GTPases in Jurkat cells treated with **4**, celastrol, and CGP37157 (Figure S8), effect of GTPase inhibitors on the cell death induced by **4** and CGP37157 (Figure S9), MTT assay of MFN1- and/or MFN2-KD Jurkat cells treated with **4** and CGP37157 (Figure S10), effect of incubation time of siRNA on the cell death in Jurkat cells treated with **4** and CGP37157 (Figure S11), MTT assay of MFN1 or MFN2 knockdown of Jurkat cells treated with celastrol (Figure S12), and chemical structures of inhibitors (Charts S1–S3) (PDF)
