# Supplementary material for: Induction of Paraptosis by Cyclometalated Iridium Complex-Peptide Hybrids and CGP37157 via a Mitochondrial Ca2+ Overload Triggered by Membrane Fusion between Mitochondria and the Endoplasmic Reticulum
Source: Biochemistry. 2022 Apr 1;61(8):639–55. doi: 10.1021/acs.biochem.2c00061 (PMC9022229; doi:10.1021/acs.biochem.2c00061)
Supplement: Supplementary file 1 — bi2c00061_si_001.pdf [file bi2c00061_si_001.pdf]

Supporting Information

Induction of Paraptosis by Cyclometalated Iridium Complex-Peptide Hybrids and CGP37157 via a Mitochondrial Ca^{2+} Overload Triggered by Membrane Fusion between Mitochondria and the Endoplasmic Reticulum

Kenta Yokoi,[a] Kohei Yamaguchi,[a] Masakazu Umezawa,[b] Koji Tsuchiya,[b] and Shin Aoki,[,a,b,c]*

[a]Faculty of Pharmaceutical Sciences, Tokyo University of Science, 2641 Yamazaki, Noda, Chiba 278-8510, Japan,

[b]Research Institute for Science and Technology (RIST), Tokyo University of Science, 2641 Yamazaki, Noda, Chiba 278-8510, Japan,

[c]Research Institute for Biomedical Science (RIBS), Tokyo University of Science, 2641

Yamazaki, Noda, Chiba 278-8510, Japan

*Corresponding authors: E-mail, shinaoki@rs.tus.ac.jp

Address of home page: http://www.rs.noda.tus.ac.jp/aokilab/

Contents

Figure S1. Effect of 2-APB, RuRed, and ER-000444793 on cell death induced by **4** evaluated by microscopic observations……………………………………………………………S3

Figure S2. Effect of 2-APB, RuRed, and ER-000444793 on cell death induced by celastrol evaluated by MTT assay……………………………………………………………………S4

Figure S3. MTT assay of HeLa S3 and A549 cells treated with CGP37157………………..S4

Figure S4. Effect of Z-VAD-fmk, necrostatin-1, and 3-methyladenine on cell death induced by CGP37157 evaluated by MTT assay…………………………………………………S5

Figure S5. Confocal microscopic observations of Jurkat cells treated with **4**, CGP37157, and celastrol and stained with MitoTracker Green and ER-Tracker Red……………………S6

Figure S6. Confocal microscopic observations of Jurkat cells treated with **4**……………S7

Figure S7. Emission intensity profiles of MitoTracker Green and ER-Tracker Red in Figure 8……………………………………………………………………………………S8

Figure S8. Western blot analysis of MFN1, MFN2 and DRP1 in Jurkat cells treated with **4**, celastrol, and CGP37157……………………………………………………………S10

Figure S9. Effect of dynasore, Mdivi-1, and CID1067700 on cell death induced by **4** and

CGP37157 evaluated by MTT assay…………………………………………..……......……S11

Figure S10. MTT assay of MFN1- and/or MFN2-KD Jurkat cells treated with **4** and CGP37157…………………………………………………………………………………S12

Figure S11. Effect of incubation time of siRNA for MFNs on the death induced by **4** and CGP37157 evaluated by Western blot analysis and MTT assay………………………………….……………………………S13

Figure S12. MTT assay of MFN1- or MFN2-KD Jurkat cells treated with celastrol…….S14

Chart 1. The structures of 2-APB, RuRed, and ER-000444793…………………………..S15

Chart 2. The structures of Z-VAD-fmk, necrostatin-1, and 3-methyladenine……………....S15

Chart 3. The structures of dynasore, Mdivi-1, and CID1067700…………………………S15

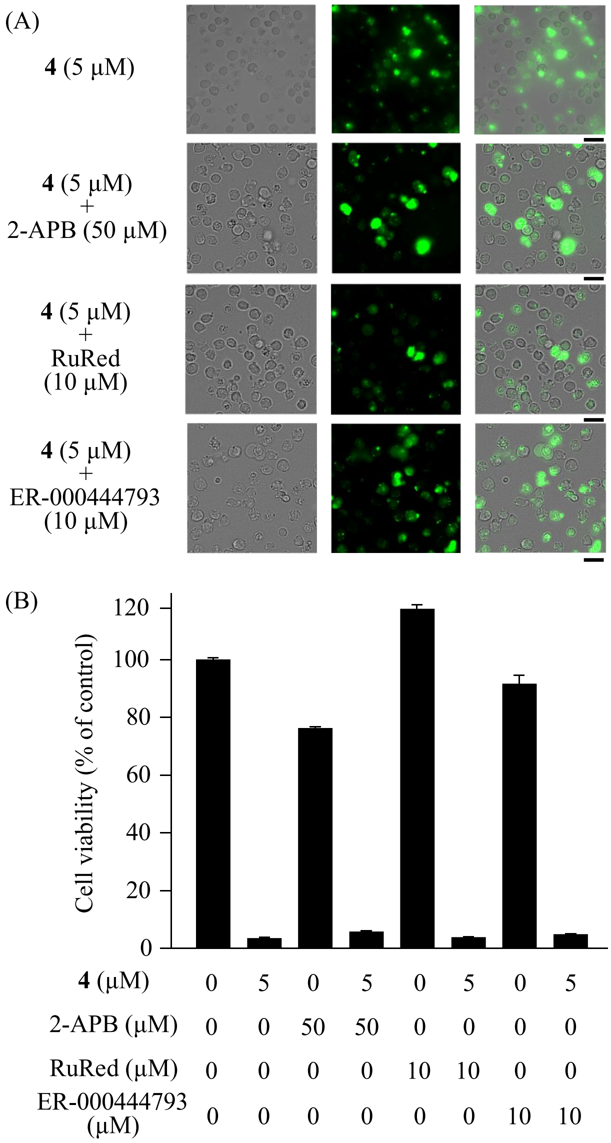

Figure S1. Effect of 2-APB (an inhibitor of the inositol 1,4,5-trisphosphate (IP_3) receptor), RuRed (an inhibitor of the mitochondrial calcium (Ca^{2+}) uniporter (MCU)), and ER-000444793 (an inhibitor of the mitochondrial permeability transition pore (mPTP)). (A) Microscopic observations of Jurkat cells pretreated with 2-APB (50 μM), RuRed (10 μM), or ER-000444793 (10 μM) at 37 °C under 5% CO_2 atmosphere for 1 h, followed by the treatment with **4** (5 μM) at 37 °C under 5% CO_2 atmosphere for 3 h in 10% FBS/RPMI medium. Excitation at 377 nm for **4** was used. Scale bar (black): 20 μm. (B) MTT assay of Jurkat cells pretreated with 2-

APB (50 μM), RuRed (10 μM), ER-000444793 (10 μM) at 37 °C under 5% CO_2 atmosphere for 1 h, followed by the treatment with **4** (5 μM) at 37 °C under a 5% CO_2 atmosphere for 3 h 10% FBS/RPMI medium.

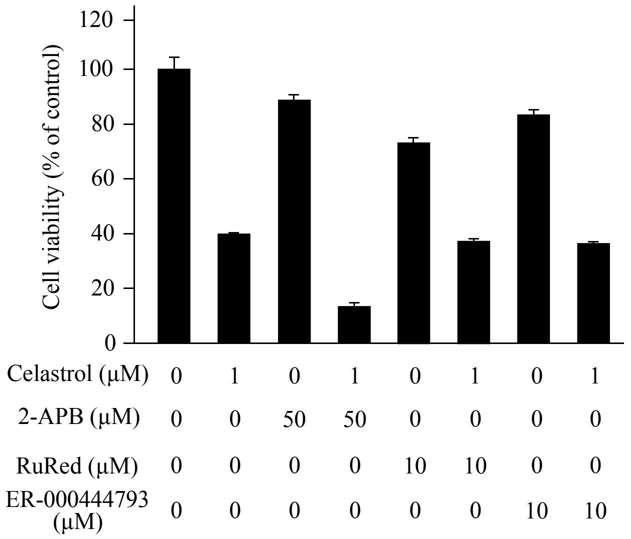

Figure S2. Effect of 2-APB, RuRed, ER-000444793 on the cell death induced by celastrol. MTT assay of Jurkat cells pretreated with 2-APB (50 μM), RuRed (10 μM), or ER-000444793 (10 μM) at 37 °C under a 5% CO_2 atmosphere for 1 h, followed by treatment with celastrol (1 μM) at 37 °C under a 5% CO_2 atmosphere for 12 h in 10% FBS RPMI medium.

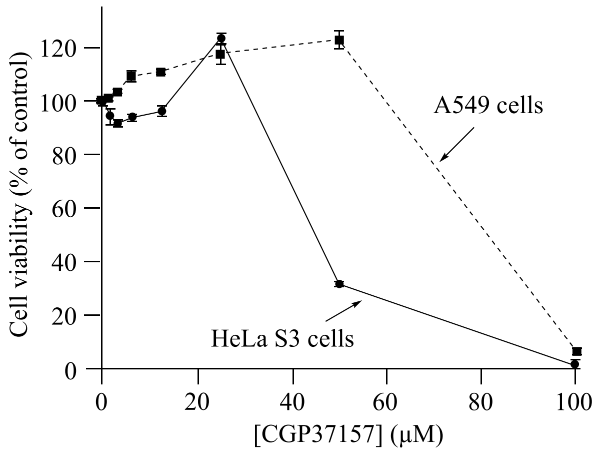

Figure S3. MTT assay of HeLa S3 cells (plain curves with filled circles) and A549 cells (dashed curves with filled squares) treated with CGP37157 (0–100 μM) in 10% FBS/MEM (HeLa S3 cells) and 10% FBS/DMEM (A549 cells) for 24 h at 37 °C under 5% CO_2.

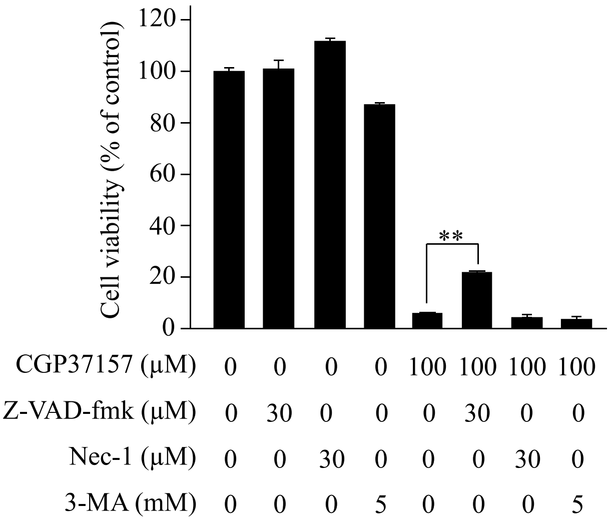

Figure S4. MTT assay of Jurkat cells treated with inhibitors of programmed cell death; Z-VAD-fmk (an apoptosis inhibitor), necrostatin-1 (Nec-1: a necroptosis inhibitor), and 3-methyl adenine (3-MA, an autophagy inhibitor) in the presence of CGP37157 (100 μM, 12 h). Values are shown as the mean ± SD for three independent experiments. ** $P < 0.01$.

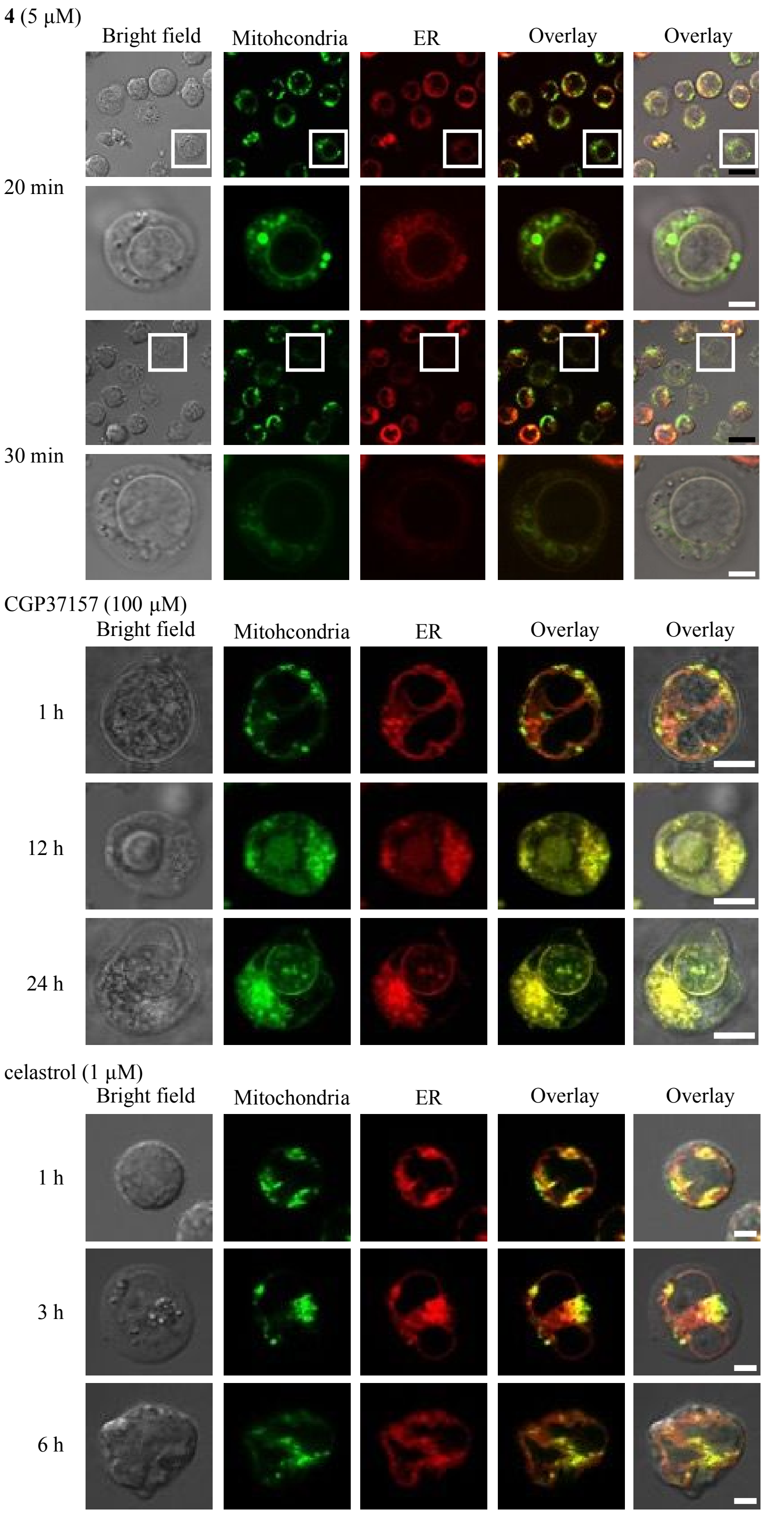

Figure S5. Confocal microscopic observations of Jurkat cells treated with **4** (5 μM), CGP37157 (100 μM), and celastrol (1 μM) in 10% FBS/RPMI medium at 37 °C under 5% CO_2 atmosphere and stained with MitoTracker Green (0.5 μM, 1 h) and ER-Tracker Red (1 μM, 1

h). Excitation at 473 nm and emission from 485–545 nm was used for MitoTracker Green. Excitation at 559 nm and emission from 570–620 nm was used for ER-Tracker Red. Exposure time was 20 μs/pixel. Scale bar (white) is 5 μm.

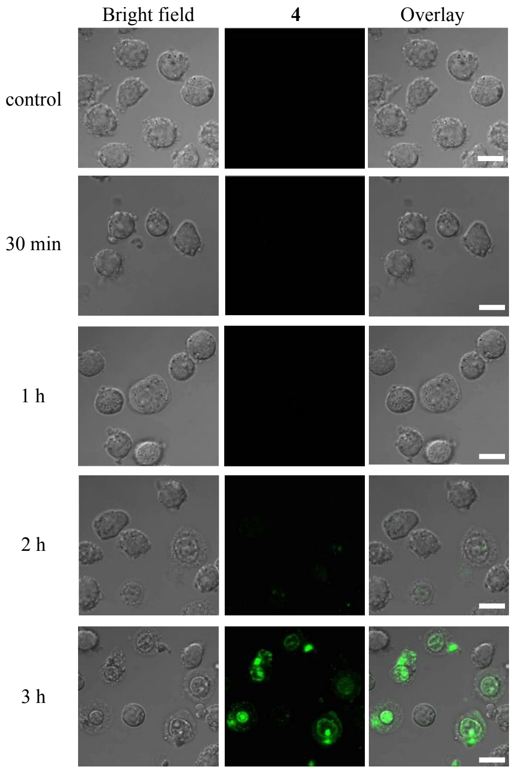

Figure S6. Confocal microscopic observations of Jurkat cells treated with **4** (5 μM) in 10% FBS/RPMI medium for 0-3 h at 37 °C under 5% CO_2 atmosphere. Excitation at 473 nm and emission from 485–545 nm was used. Scale bar (white) is 10 μm.

(a) control (from Figure 8d in the text)

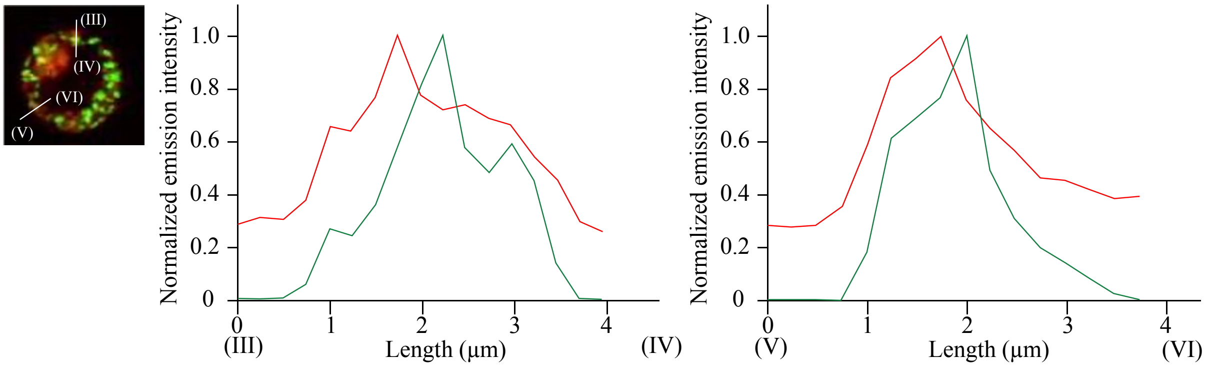

(b) **4** (5 μM, 10 min) (from Figure 8i in the text)

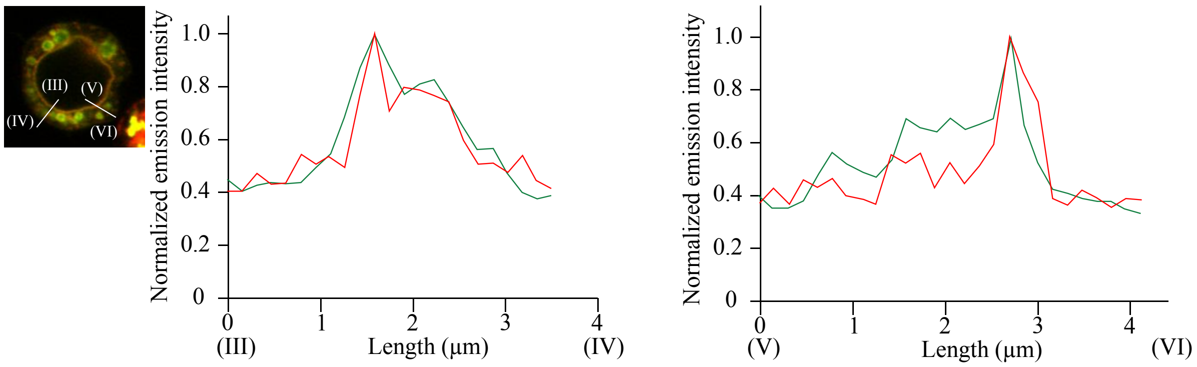

(c) control (from Figure 8n in the text)

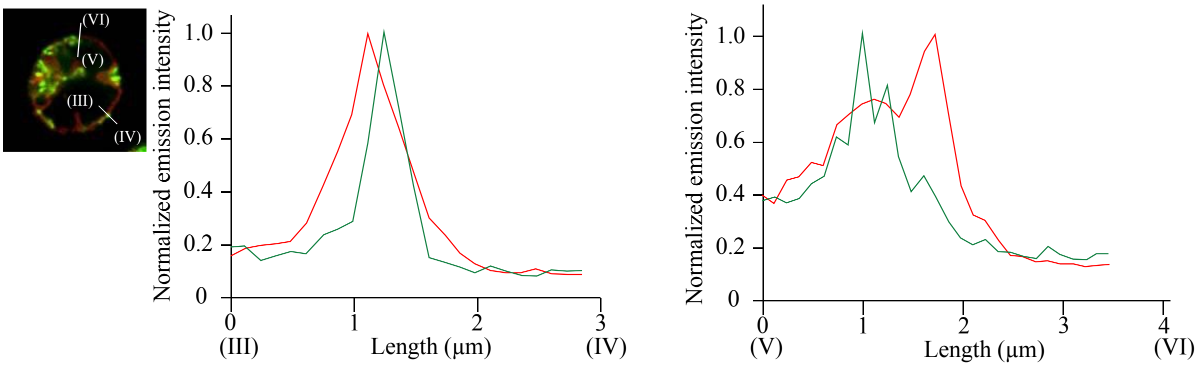

(d) CGP37157 (100 μM, 6 h) (from Figure 8s in the text)

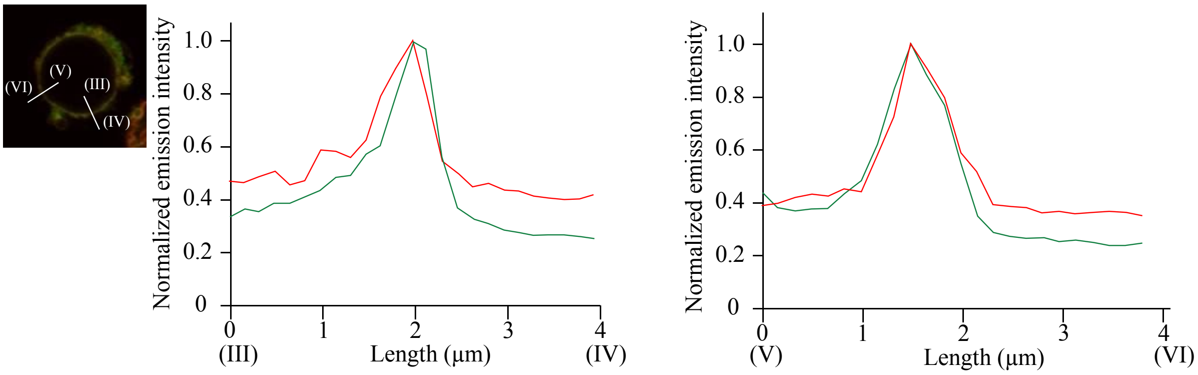

(e) control (from Figure 8x in the text)

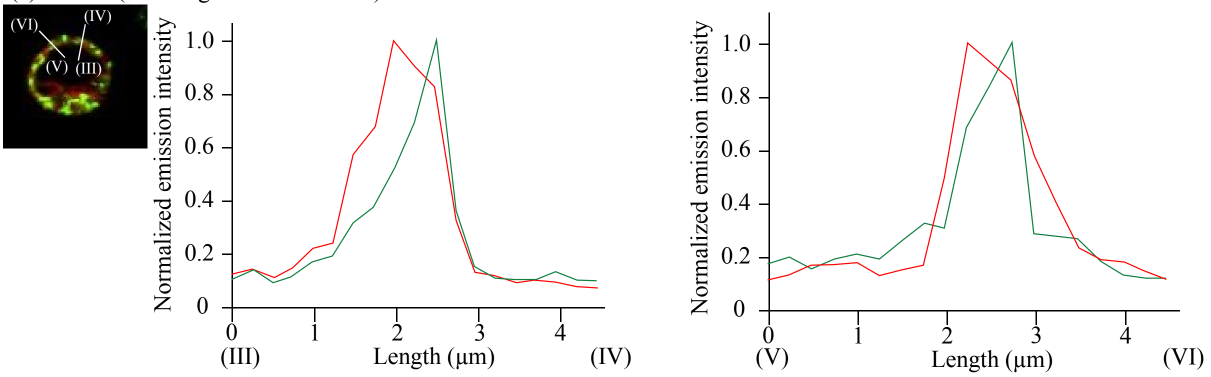

(f) celastrol (1 μM, 12 h) (from Figure 8ac in the text)

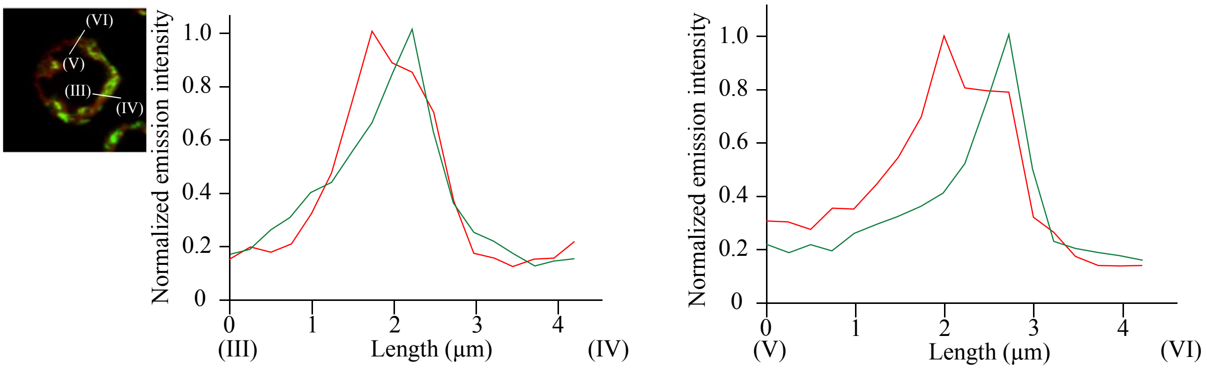

Figure S7. Emission intensity profiles of MitoTracker Green (green) and ER-Tracker Red (red) from the point (III) to (IV) or (V) to (VI) in Figure S7a (same as Figure 8d in the text), Figure S7b (same as Figure 8i in the text), Figure S7c (same as Figure 8n in the text), Figure S7d (same as Figure 8s in the text), Figure S7e (same as Figure 8x in the text), and Figure S7f (same as Figure 8ac in the text).

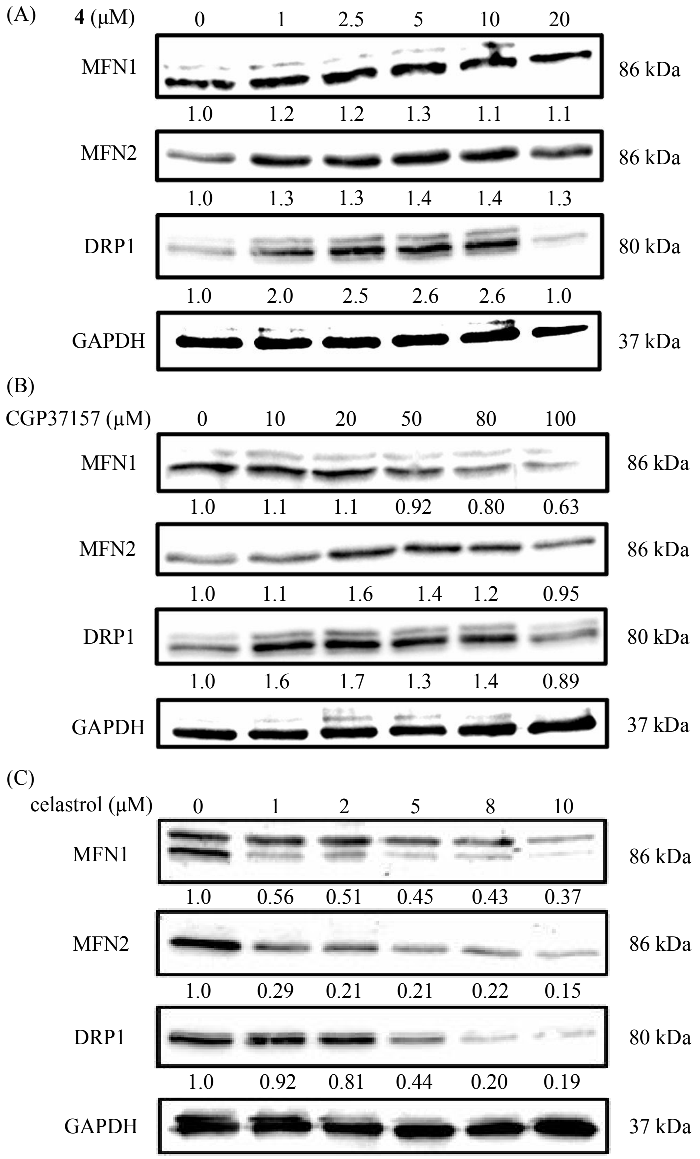

Figure S8. Western blot analyses of mitofusin 1 (MFN1), mitofusin 2 (MFN2), and dynamin-related protein 1(DRP1) in Jurkat cells (A) treated with **4** (0–5 μM) for 1 h, (B) treated with CGP37157 (0–100 μM) for 12 h, and (C) treated with celastrol (0–10 μM) for 24 h at 37 °C under 5% CO_2. The intensity of each band was compared based on the condition in the absence of **4**, celastrol, and CGP37157 and the values are shown ender the bands.

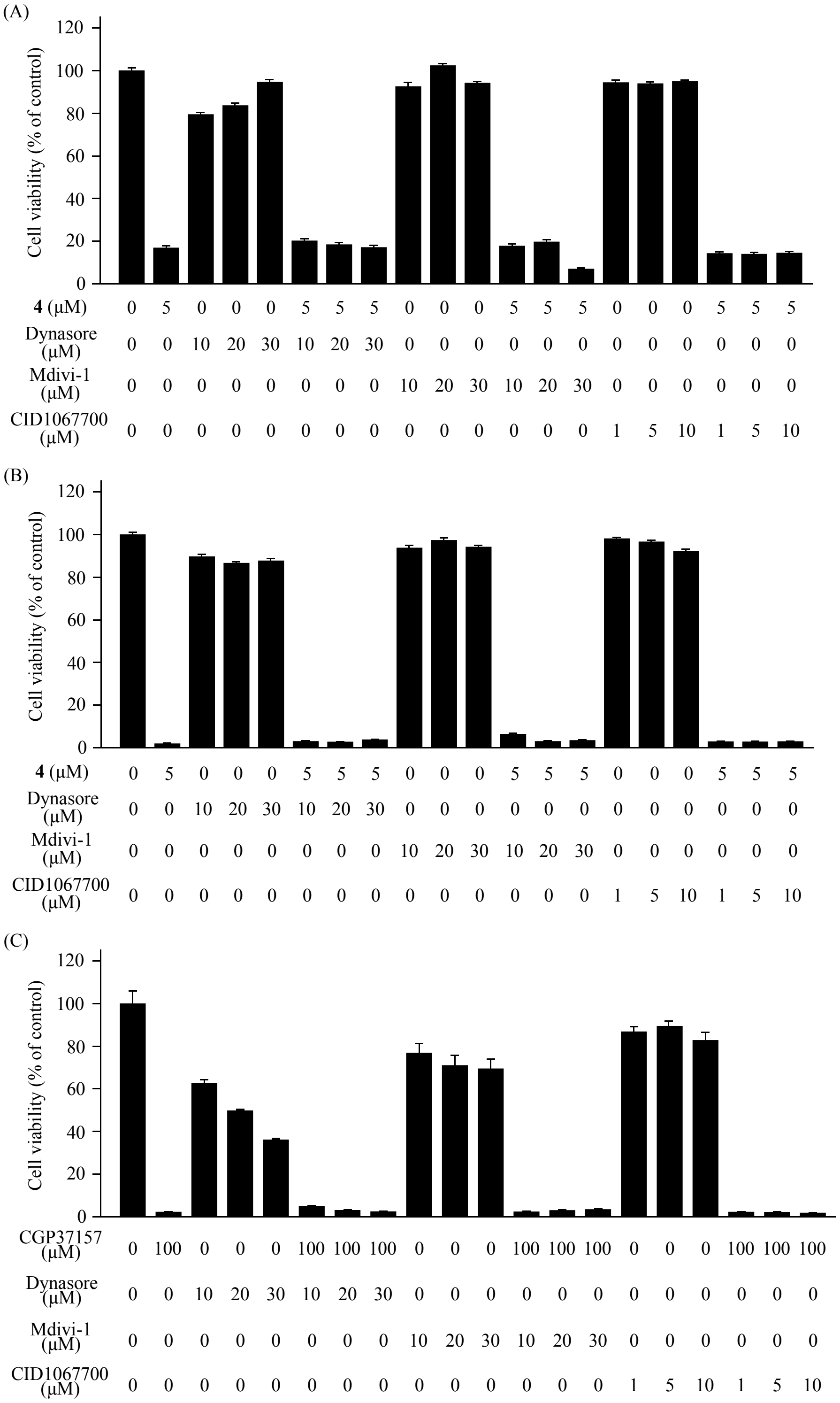

Figure S9. Effect of dynasore (an inhibitor of dynamin-1, -2, and dynamin-related protein 1 (DRP1)), mitochondrial division inhibitor 1 (Mdivi-1: a selective inhibitor of DRP1), and CID1067700 (a competitive inhibitor of Ras-related GTPase) on the cell death of Jurkat cells

induced by **4** (5 μM) for (A) 1 h or (B) 3 h and (C) CGP37157 (100 μM) for 12 h, as evaluated by MTT assay. Values are the mean ± SD for three independent experiments.

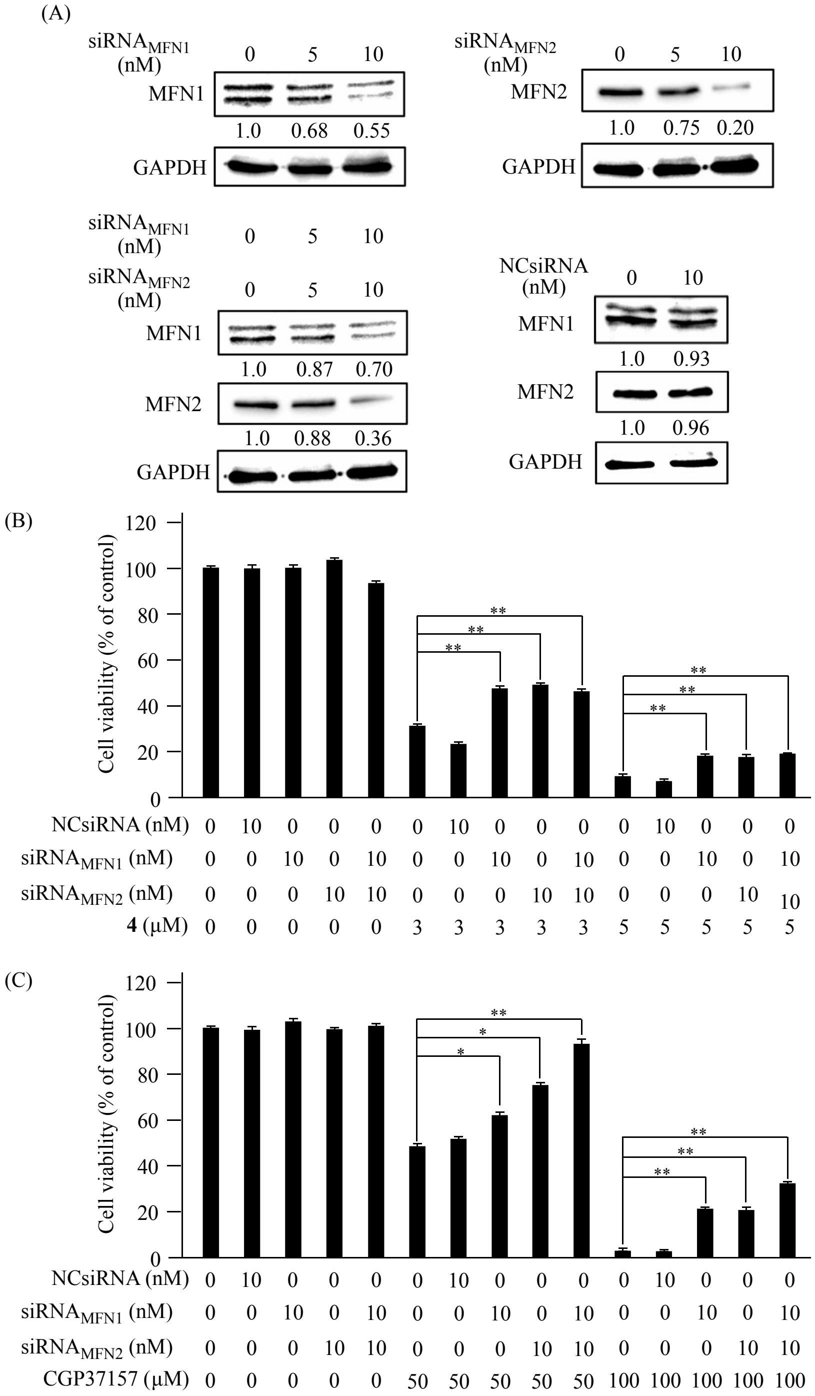

Figure S10. (A) Western blot analyses of MFNs in Jurkat cells treated with $siRNA_{MFN1}$ and/or

$siRNA_{MFN2}$, or negative control siRNA (NCsiRNA). The intensity of each band was compared based on the condition in the absence of siRNA, and the values are shown under the bands. (B) MTT assay of MFN1- and/or MFN2-KD Jurkat cells treated with **4** (3 or 5 μM) at 37 °C under 5% CO_2 for 1 h. (C) MTT assay of MFN1- and/or MFN2-KD Jurkat cells treated with CGP37157 (50 or 100 μM) at 37 °C under 5% CO_2 for 12 h. Values are shown as the mean ± SD for three independent experiments. *: $P < 0.05$, **: $P < 0.01$.

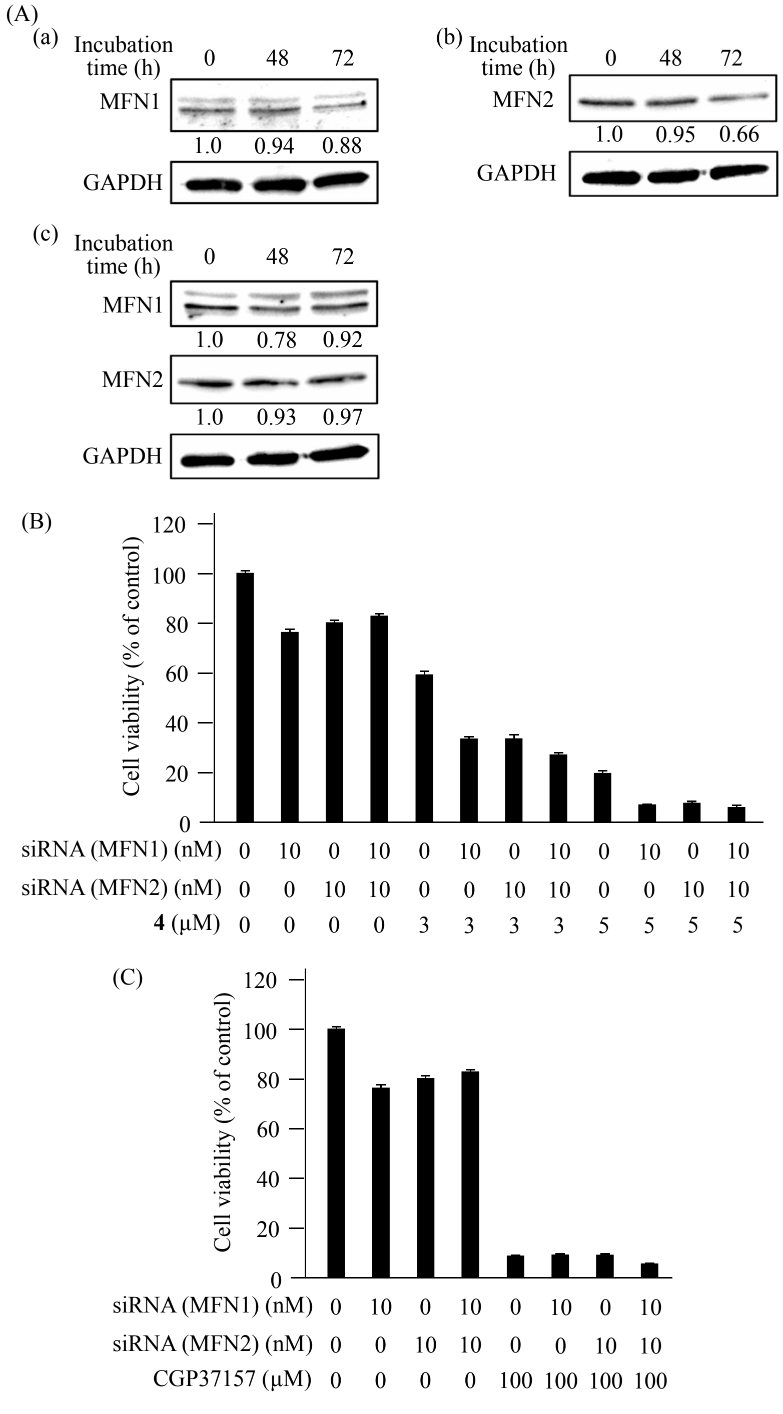

Figure S11. (A) Western blot analysis of MFNs in Jurkat cells treated with siRNA for (a) MFN1, (b) MFN2, and (c) MFN1 and MFN2 (5 nM) at 37 °C under 5% CO_2 for 48 or 72 h. The intensity of each band was compared based on the condition in the absence of siRNA, and the values are shown under the bands. (B) MTT assay of MFN1- and/or MFN2-KD Jurkat

cells treated with **4** (3 or 5 μM) at 37 °C under 5% CO_2 for 1 h. (C) MTT assay of MFN1- and/or MFN2-KD Jurkat cells treated with CGP37157 (100 μM) at 37 °C under 5% CO_2 for 12 h. Values are shown as mean ± SD for three independent experiments.

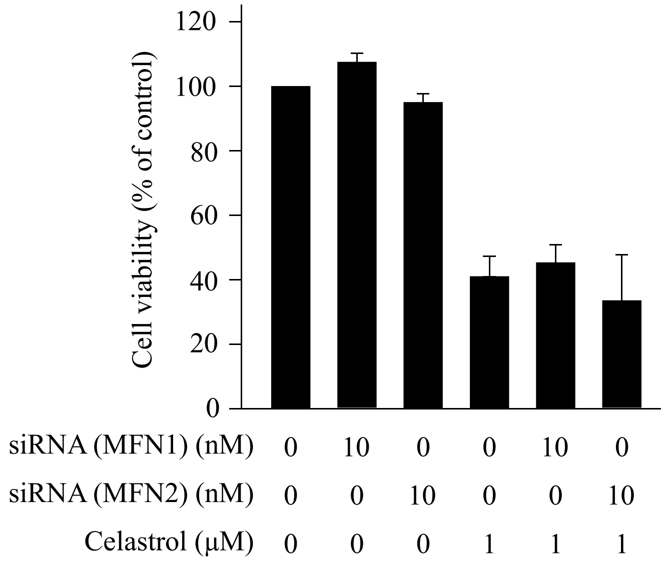

Figure S12. MTT assay of MFN1-KD and MFN2-KD Jurkat cells treated with celastrol (1 μM) at 37 °C under 5% CO_2 for 12 h. Values are shown as mean ± SD for three independent experiments.

Chart S1. The structures of 2-APB, RuRed, and ER-000444793

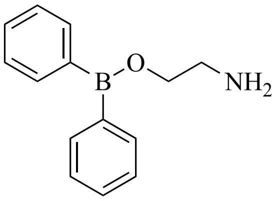

2-Aminoethoxydiphenyl borate
(2-APB)

$[Ru(NH_3)_6\text{-O-}Ru(NH_3)_4\text{-O-}Ru(NH_3)_6]^{6+}$
6Cl-

RuRed

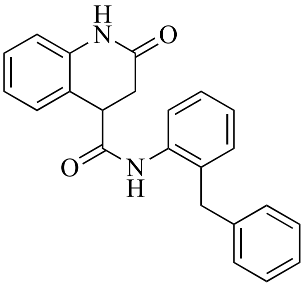

ER-000444793

Chart S2. The structures of Z-VAD-fmk, necrostatin-1, and 3-methyladenine

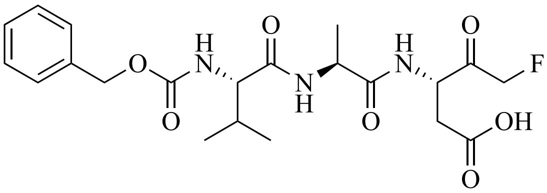

Z-VAD-fmk

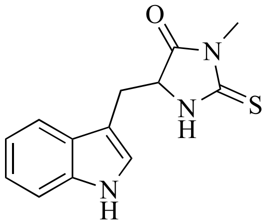

necrostatin-1

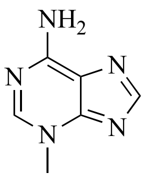

3-methyladenine

Chart S3. The structures of dynasore, Mdivi-1, and CID1067700

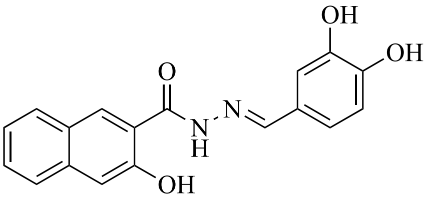

Dynasore

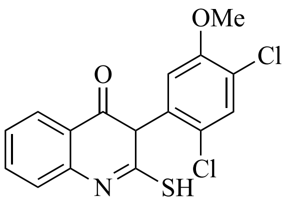

Mdivi-1

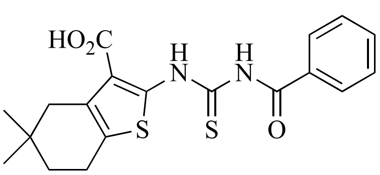

CID1067700
